# Surface reconstruction of metal halides for S-site shielding toward long-cycle all-solid-state lithium batteries

**DOI:** 10.1039/d6sc03904a

**Published:** 2026-06-30

**Authors:** Wenrui Hu, Qingkun Zhu, Fenghua Zheng, Xinyou He, Heng Wen, Zhiming Xiao, Wei Xu, Fenghua Ding, Xinghui Liang, Lei Ming, Baishan Chen, Changsheng An, Zhang Lin, Xing Ou

**Affiliations:** a Engineering Research Center of the Ministry of Education for Advanced Battery Materials, School of Metallurgy and Environment, Central South University Changsha 410083 P. R. China liangxh@csu.edu.cn ouxing@csu.edu.cn; b Guangxi Key Laboratory of Low Carbon Energy Materials, School of Chemistry and Pharmaceutical Sciences, Guangxi Normal University Guilin 541004 P. R. China; c Guangxi Huayou Lithium Industry Co., Ltd Yulin 537624 China; d Institute for Advanced Study, Central South University Changsha 410083 China 221333@csu.edu.cn; e School of Materials and Environmental Engineering, Changsha University Changsha 410022 P. R. China z20190628@ccsu.edu.cn

## Abstract

Despite their high ionic conductivity and mechanical processability, sulfide electrolytes suffer from severe interfacial reactivity and structural incompatibility with Ni-rich layered oxide cathodes, causing rapid capacity fading that limits their practical viability in all-solid-state lithium batteries. Conventional coatings provide primarily physical isolation without fully eliminating reactive surface sulfur sites, rendering the interface vulnerable to gradual deterioration over prolonged cycling. Here, we propose a coordination-driven interfacial reconstruction strategy that exploits the volatility and strong Lewis acidity of ZrCl_4_ to enable its interaction with electron-rich sulfur species on the Li_6_PS_5_Cl surface, thereby inducing the *in situ* formation of a Li–Zr–Cl–S (LZCS) passivation interphase. The resulting protective interphase exhibits favorable chemical compatibility with the sulfide host and effectively passivates highly reactive surface sulfur sites, thereby substantially suppressing parasitic interfacial reactions. As a result, the cell pairing the modified electrolyte with LiNi_0.8_Co_0.1_Mn_0.1_O_2_ (NCM811) cathode retains 91.4% capacity after 1400 cycles at 1C, highlighting exceptional long-cycle stability under practical conditions. Even at a high cutoff voltage of 4.5 V, the cell still exhibits 94.5% capacity retention after 600 cycles (1C). This work establishes a strategy for constructing functional halide interphases on sulfide electrolytes, thereby enabling stable integration with Ni-rich layered cathodes.

## Abstract 2

This work uses ZrCl_4_ to reconstruct the Li_6_PS_5_Cl surface and form a stable Li–Zr–Cl–S passivation layer, improving the compatibility between sulfide electrolytes and Ni-rich cathodes.

## Graphical abstract

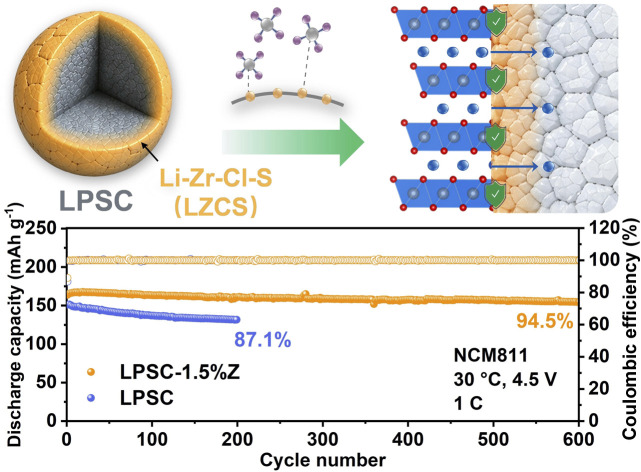

## Introduction

All-solid-state lithium batteries (ASSLBs) are widely regarded as a promising next-generation energy storage technology owing to their superior safety and high energy density.^1,2^ ASSLBs replace liquid electrolytes with nonflammable solid electrolytes, fundamentally eliminating leakage and safety risks.^3,4^ Among various solid electrolytes, sulfide electrolytes are widely studied owing to their high ionic conductivity, approaching that of liquid electrolytes, and their good mechanical deformability. These advantages make them strong candidates for constructing high-energy-density ASSLBs.^5–8^ In particular, Li_6_PS_5_Cl (LPSC), a representative argyrodite-type sulfide electrolyte, combines a moderate cost advantage with relatively good electrochemical stability, making it one of the most promising candidates for the large-scale application of ASSLBs.^9,10^

Notably, the pursuit of high energy density relies on Ni-rich layered oxide cathodes, typified by LiNi_*x*_Co_*y*_Mn_1−*x*−*y*_O_2_ (NCM) and LiNi_*x*_Co_*y*_Al_1−*x*−*y*_O_2_ (NCA).^11–14^ However, direct contact with sulfide electrolytes can introduce pronounced interfacial incompatibility, as the sulfur species (S^2−^) in the LPSC are susceptible to oxidation, generating electron- and ion-insulating by-products, while the PS_4_^3−^ framework also undergoes concurrent degradation.^15,16^ In addition, S^2−^ can react with high-valent transition metals in the cathode (Ni^4+^ and Co^4+^), disrupting the layered structure of the cathode.^17^ These interfacial reactions lead to the accumulation of decomposition products, the formation of pores and microcracks, and local volume changes, all of which hinder Li^+^ transport and accelerate capacity decay.^18^ Accordingly, surface coating has been widely recognized as an effective strategy for mitigating such interfacial degradation.^19–22^ Among the available coating materials, halide electrolytes are particularly attractive interfacial modifiers because they combine superior oxidative stability with favorable compatibility toward oxide cathodes.^23^ When introduced at the cathode/electrolyte interface, halide phases can physically decouple sulfide electrolytes from the cathode, thereby protecting reactive sulfur species from oxidation-induced decomposition.^24^ Meanwhile, their relatively high Li^+^ conductivity allows interfacial stabilization to be achieved without excessively compromising ionic transport.^25,26^ However, halide coatings still face a common issue shared by most coating strategies, namely the introduction of a new halide/sulfide heterointerface. In most cases, such a simple physically isolated interface cannot fully block the highly reactive S sites, and particularly when the coating is uneven, the interface may undergo degradation or failure during long-term cycling. Therefore, it is crucial to develop a uniformly distributed halide electrolyte coating that can chemically suppress the reactivity of S species.^27,28^

Conventional methods for constructing interfacial layers mainly include solid-phase grinding and liquid-phase coating, which still struggle to simultaneously meet the above requirements.^29^ Solid-phase methods are simple, but conventional ball milling often results in uneven surface coverage and may damage the sulfide electrolyte, reducing ionic conductivity. Liquid-phase methods can improve coating homogeneity, but the high solvent sensitivity of sulfide electrolytes means that improper solvent selection may induce material decomposition,^30^ disrupt the interfacial chemical environment, and deteriorate electrochemical performance.^31,32^ Given its potential for creating uniform interfaces, vapor-phase coating has attracted significant attention, with recent efforts utilizing HF,^33^ HCl,^34^ and CO_2_ (ref. 35) to functionalize electrolyte surfaces in a controlled manner. However, these layers still primarily provide spatial blocking, without altering the chemical reactivity of the S sites. Moreover, most halide electrolytes possess low volatility and limited vapor-phase reactivity, making effective deposition and interface construction on sulfide electrolytes challenging. Therefore, developing a new strategy to build a uniformly passivating interphase remains an urgent task.

**Fig. 1:**
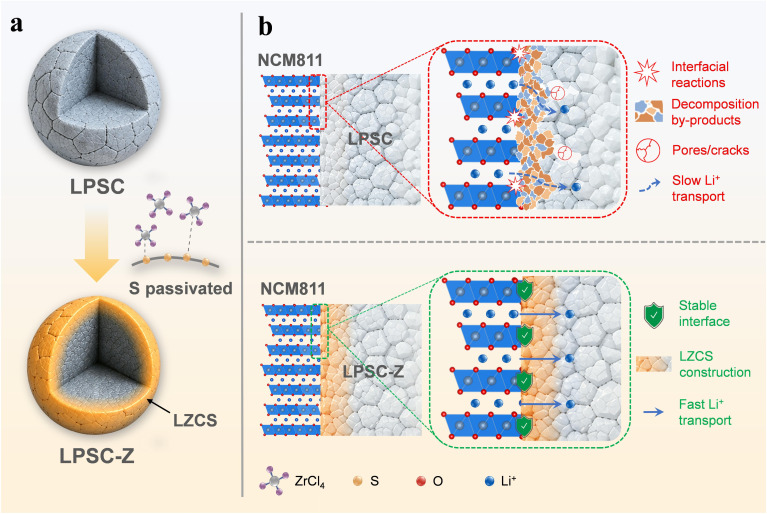
(a) Schematic of the LZCS passivation layer construction and (b) the corresponding interfacial mechanism in ASSLBs.

Based on the above considerations, this work proposes a coordination-driven interfacial reconstruction strategy. This strategy exploits the volatility and strong Lewis acidity of ZrCl_4_, enabling its vapor-phase introduction and coordination with electron-rich sulfur species on the LPSC surface to form a stable Li–Zr–Cl–S (LZCS) halide layer composed of Zr–S and Zr–Cl species, together with LiCl (Fig. 1a). The *in situ* formed LZCS layer not only provides physical separation at the cathode/sulfide electrolyte interface but also, more importantly, chemically passivates the highly reactive S sites, thereby effectively suppressing undesirable side reactions. Consequently, the modified LPSC exhibited enhanced mechanical and chemical stability, enabling outstanding electrochemical performance in the matched ASSLBs (Fig. 1b). NCM811 half-cells assembled with the LZCS-passivated electrolyte exhibit a capacity retention of 91.4% after 1400 cycles at 1C, significantly outperforming their pristine counterpart (76.1%). Even under a high cutoff voltage of 4.5 V, a remarkable capacity retention of 94.5% is exhibited after 600 cycles. This coordination-driven *in situ* interfacial reconstruction strategy represents a valuable advancement and broadens the design strategy for regulating the interfacial reactivity of sulfide electrolytes against Ni-rich layered cathodes.

## Results and discussion

**Fig. 2:**
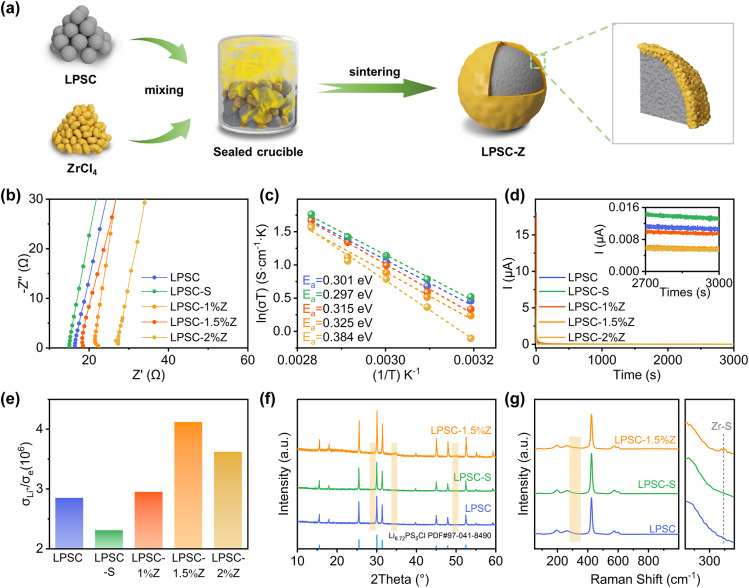
Preparation and transport-selectivity screening of ZrCl_4_ modified LPSC toward the optimal composition. (a) Schematic illustration of the preparation process of ZrCl_4_ treated LPSC. (b) Ionic conductivities, (c) apparent activation energies for ionic conduction, (d) DC polarization curves and (e) ionic-to-electronic conductivity ratios (*σ*_Li^+^_/*σ*_e_) of pristine LPSC, LPSC-S, and ZrCl_4_ treated LPSC samples with different ZrCl_4_ contents. (f) XRD patterns and (g) Raman spectra of pristine LPSC, LPSC-S, and LPSC-1.5%Z.

The synthesis strategy is schematically illustrated in Fig. 2a. LPSC powder was mixed with a designed amount of ZrCl_4_ and heat-treated in a sealed glass crucible subsequently. Upon heating, ZrCl_4_ sublimated and was transported to the surface of LPSC particles through a vapor-phase process, leading to surface modification of LPSC. By adjusting the ZrCl_4_ content to 1.0, 1.5, and 2.0% by weight relative to LPSC, a series of samples with different modification degrees were obtained, denoted as LPSC-1%Z, LPSC-1.5%Z, and LPSC-2%Z, respectively. To distinguish the effect of ZrCl_4_ treatment from that of sintering alone, a control sample was prepared by subjecting pristine LPSC to the same thermal protocol in the absence of ZrCl_4_, denoted as LPSC-S. As shown in Fig. S1, the ZrCl_4_ treated samples changed in color from gray to light yellow, suggesting an altered surface chemistry after treatment.

As shown in Fig. 2b and S2, pristine LPSC exhibited an ionic conductivity of 4.63 mS cm^−1^, which slightly increased to 4.74 mS cm^−1^ after thermal treatment (LPSC-S), indicating that the heat-treatment protocol itself exerted only a minor influence on Li^+^ transport. After ZrCl_4_ treatment, the ionic conductivities of LPSC-1%Z, LPSC-1.5%Z, and LPSC-2%Z decreased to 4.11, 3.55, and 2.79 mS cm^−1^, respectively. To further evaluate the effect of surface modification on Li^+^ transport, the apparent activation energy for ionic conduction was derived from temperature-dependent conductivity measurements (Fig. 2c).^36^ Pristine LPSC exhibited an activation energy of 0.301 eV, which increased to 0.315, 0.325, and 0.384 eV for LPSC-1%Z, LPSC-1.5%Z, and LPSC-2%Z, respectively, indicating that ZrCl_4_ treatment moderately increased the Li^+^ transport barrier. Taken together, the decreased ionic conductivity and increased activation energy indicated that excessive coating, as in LPSC-2%Z, was unfavorable for Li^+^ transport.

In addition to ionic conductivity, the electronic conductivity of the samples was further evaluated by DC polarization. As shown in Fig. 2d and S3, all samples rapidly reached steady-state currents with low plateau values during polarization, indicating their intrinsically low electronic conductivity. Compared with pristine LPSC, LPSC-S exhibited a slightly higher electronic conductivity, increasing from 1.62 × 10^−6^ to 2.05 × 10^−6^ mS cm^−1^, which was attributed to heat treatment induced microstructural evolution, particularly grain growth and improved interparticle contact.^37^ In contrast, the steady-state current progressively decreased with increasing ZrCl_4_ content, confirming that the surface-modified layer effectively suppressed electronic conduction.

To comprehensively assess the transport selectivity of the modified electrolytes, the ratio of ionic to electronic conductivity (*σ*_Li^+^_/*σ*_e_) was compared, as shown in Fig. 2e. Although pristine LPSC exhibited high ionic conductivity, its *σ*_Li^+^_/*σ*_e_ ratio remains limited, indicating insufficient ionic selectivity. Among all samples, LPSC-1.5%Z exhibited the highest *σ*_Li^+^_/*σ*_e_ ratio, showing an ionic conductivity of 3.55 mS cm^−1^ while lowering the electronic conductivity to 8.62 × 10^−7^ mS cm^−1^. These results demonstrated that 1.5 wt% ZrCl_4_ provided the most favorable balance between fast Li^+^ conduction and suppressed electronic leakage, and this composition was therefore selected for subsequent characterization and evaluation.

To preliminarily predict the possible interaction between ZrCl_4_ and the LPSC surface, DFT calculations were performed, as shown in Fig. S4. Owing to the strong affinity between Zr^4+^ and S^2−^, sulfur-related species on the LPSC surface can react with ZrCl_4_, accompanied by partial replacement of Cl atoms by surface sulfur species.^38^ The calculated energy change after this reaction was negative, with a decrease of 7.15 eV, indicating that the reaction between ZrCl_4_ and sulfur-containing sites on the LPSC surface was energetically favorable.^39^ Notably, the optimized structure showed the formation of Zr–S coordination while part of the Zr–Cl bonding environment is retained. To probe the structural evolution induced by ZrCl_4_ treatment, X-ray diffraction (XRD) was performed, as shown in Fig. 2f. The diffraction peaks of the pristine sample were well indexed to cubic argyrodite Li_6.72_PS_5_Cl, confirming the successful synthesis of phase-pure LPSC. Notably, no additional diffraction peaks were observed after the secondary heat treatment at 400 °C (LPSC-S), indicating that the thermal treatment did not induce detectable impurity phases. After ZrCl_4_ treatment, the XRD pattern still largely retained the characteristic framework reflections of LPSC, indicating that the bulk framework remained generally stable. Meanwhile, local peak-shape variations were observed in the marked region, where the diffraction peaks became evidently broadened. These broadened features indicated reduced crystallinity and partial amorphous characteristics in the corresponding region, suggesting that ZrCl_4_ treatment induces surface reconstruction of LPSC. Raman spectroscopy was further employed to probe local structural changes in the modified electrolyte (Fig. 2g). All samples exhibited a strong characteristic band at around 420 cm^−1^, corresponding to the vibrational mode of PS_4_^3−^ tetrahedral units, further indicating that the LPSC host framework was largely preserved after treatment.^40^ Notably, an additional feature emerged at around 320 cm^−1^ for LPSC-1.5%Z, which originated from a newly formed local Zr–S coordination environment induced by ZrCl_4_ treatment.^41^

**Fig. 3:**
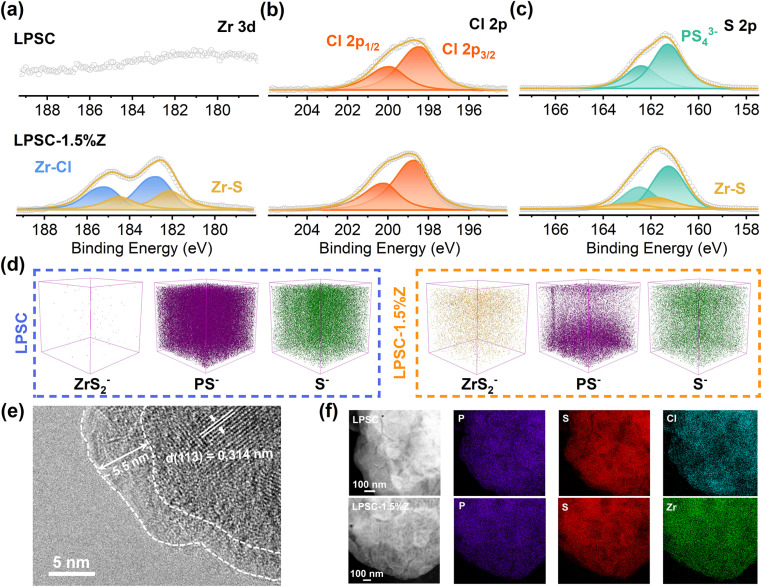
XPS and TEM characterization of LPSC and LPSC-1.5%Z. (a) XPS spectra of Zr 3d, (b) Cl 2p, and (c) S 2p for LPSC and LPSC-1.5%Z. (d) TOF-SIMS of LPSC and LPSC-1.5%Z. (e) TEM image of LPSC-1.5%Z. (f) EDS mapping of LPSC and LPSC-1.5%Z.

To further elucidate the composition and surface chemical states of the reconstructed interphase, X-ray photoelectron spectroscopy (XPS) was performed. As shown in Fig. 3a–c, no Zr signal was detected for pristine LPSC, whereas LPSC-1.5%Z exhibited a pair of characteristic peaks at 182.7 eV and 185.2 eV in the Zr 3d region, which were attributed to the Zr–Cl bonds, indicating the successful introduction of Zr species onto the surface of LPSC.^42^ Additionally, characteristic peaks corresponding to Zr–S were observed at 182.1 eV and 184.5 eV, further confirming that the introduced ZrCl_4_ reacted with the sulfur species in LPSC.^43^ In the P 2p spectra, both pristine LPSC and LPSC-1.5%Z retained the characteristic signals of PS_4_^3−^ units, suggesting that the LPSC framework remained largely preserved after modification (Fig. S5).^40^ Meanwhile, the Cl 2p spectrum of LPSC-1.5%Z shifts by about 0.2 eV toward higher binding energy compared with that of pristine LPSC. This slight shift can be attributed to the introduction of Zr–Cl coordination in ZrCl_4_, where the highly electropositive and strongly Lewis acidic Zr withdrew electron density from Cl, thereby lowering the local electron density.^37^ More importantly, in the S 2p region, LPSC-1.5%Z exhibited, in addition to the PS_4_^3−^ signals at 161.3 eV and 162.5 eV,^37^ the emergence of a new Zr–S signal at 161.8 eV and 163.0 eV.^37^ These XPS results revealed that ZrCl_4_ vapor treatment induces *in situ* surface reconstruction on LPSC, leading to the formation of a new derived interface. By contrast, the XPS spectra of thermally treated LPSC-S remained essentially identical to those of pristine LPSC (Fig. S6), indicating that short-term heat treatment alone did not markedly alter the surface chemical states.

To further verify the evolution of the surface coordination environment, time-of-flight secondary-ion mass spectrometry (TOF-SIMS) was performed (Fig. 3d and S7). For pristine LPSC, no obvious ZrS_2_^−^ signal was detected, while both PS^−^ and S^−^ exhibited relatively uniform spatial distributions, indicating the intrinsic structural uniformity of the thiophosphate framework and sulfur species in LPSC. In contrast, a clear ZrS_2_^−^ signal was observed in LPSC-1.5%Z, confirming the successful introduction of Zr and the formation of Zr–S related coordination environments. Meanwhile, the uniform S^−^ distribution in LPSC-1.5%Z further indicated that the surface treatment did not cause obvious local elemental segregation. Importantly, for the LPSC-1.5%Z sample, the ZrCl_2_^−^ and LiCl_2_^−^ fragment mapping (Fig. S8) exhibited spatial distributions highly consistent with that of ZrS_2_^−^ within the passivation region. Compared with the homogeneous LiCl_2_^−^ distribution in pristine LPSC, LPSC-1.5%Z exhibited pronounced LiCl_2_^−^ enrichment in the reconstructed surface region, consistent with localized LiCl formation. Combined with the TOF-SIMS evidence for the formation of Zr–S coordination, charge density difference calculations further revealed electron redistribution at the interface (Fig. S9). The cyan and yellow regions represented electron depletion and electron accumulation, respectively. The electron density around S species decreased, while electron accumulation was observed within the Zr–S bonding region, suggesting the stabilization of S-related surface species and enhanced interfacial chemical stability.^44,45^ These results indicated that Zr reacted with S-related species in LPSC, leading to the reconstruction of the LPSC surface into a Li–Zr–Cl–S (LZCS) passivation layer composed of Zr–S and Zr–Cl species, together with LiCl. This passivation layer played a critical role in shielding the S sites, which arose from the combined effects of chemical binding and anchoring through Zr–S coordination and the accompanying interfacial electronic redistribution.

Scanning electron microscopy (SEM) was further employed to visualize the distribution and morphology of the LZCS layer (Fig. S10). Both pristine LPSC and LPSC-1.5%Z consisted of irregular micrometer-sized particles with comparable overall morphology and particle-size distribution, suggesting that the vapor-phase treatment has little influence on the bulk particle morphology. Notably, fine surface-adhered particles were observed on LPSC-1.5%Z. SEM-EDS analysis of a representative particle decorated with these fine features (Fig. S11 and S12) revealed the coexistence of S, P, Zr, and Cl. Combined with the absence of obvious Zr-rich aggregates, this result suggested that these features originated from grain growth during the secondary annealing process rather than simple deposition of ZrCl_4_, further indicating that the passivation layer formed *via* vapor-phase introduction was uniformly distributed.

To directly visualize the interfacial structure, transmission electron microscopy (TEM) was performed on LPSC-1.5%Z. As shown in Fig. 3e, a continuous surface layer with a thickness of approximately 5.5 nm can be clearly observed on the particle surface, providing direct microscopic evidence for the successful formation of the passivation layer. No distinct long-range ordered lattice fringes were observed within the surface layer, suggesting that the passivation layer is poorly crystalline or nearly amorphous in nature. In contrast, the particle core exhibited well-defined lattice fringes with an interplanar spacing of approximately 0.314 nm, corresponding to the (113) plane of bulk LPSC, indicating that the crystallinity of the LPSC bulk phase is largely preserved after modification. Moreover, TEM-EDS elemental mapping (Fig. 3f) still confirmed the uniform distribution of Zr on the particle, whereas the P and S signals mainly originated from the LPSC host framework, further evidencing the uniformity of the surface modification. In summary, these characterization studies demonstrated that the introduced ZrCl_4_ successfully reacted with surface S species of LPSC to form a uniformly distributed LZCS passivation layer, while largely maintaining the bulk framework of LPSC. This verified that the proposed interfacial reconstruction strategy achieved effective and controllable functionalization of the LPSC surface.

**Fig. 4:**
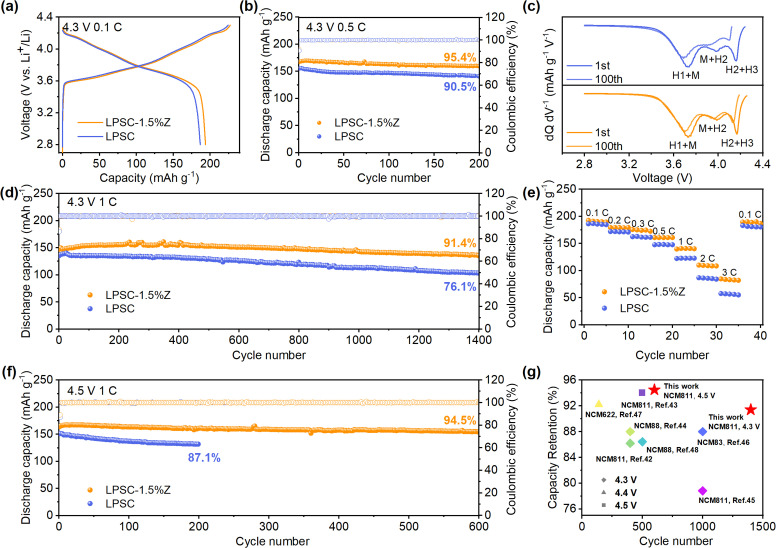
Electrochemical performance of the cells with LPSC and LPSC-1.5%Z solid electrolytes. (a) Initial charge–discharge curves. (b) Cycling performance at 0.5C. (c) Corresponding d*Q*/d*V* curves. (d) Long-term cycling performance at 1C under 4.3 V. (e) Rate performance from 0.1C to 3C. (f) Long-term cycling performance at 1C under 4.5 V. (g) Performance comparison of sulfide-based ASSLBs with the literature.

After the formation of the LZCS passivation layer, its influence on the electrochemical stability of the electrolyte was first evaluated by linear sweep voltammetry (LSV). As shown in Fig. S13, LPSC-1.5%Z exhibited a noticeably lower oxidation current than pristine LPSC, particularly around the main oxidation region near 3.41 V. The suppressed oxidation behavior indicated that the reconstructed LZCS layer effectively alleviated oxidative decomposition, thereby enhancing the electrochemical stability of the electrolyte. Then the NCM811‖LiIn cells were assembled using uncoated NCM811 cathodes (Fig. S14) and different electrolytes to evaluate their electrochemical performance.^40^ As shown in Fig. 4a and S15, when tested at 0.1C with a cutoff voltage of 4.3 V, all cells exhibit similar charge–discharge plateaus. The cells with LPSC and LPSC-S delivered initial coulombic efficiencies (ICE) of 83.1% and 83.0%, with corresponding initial discharge capacities of 186.6 and 186.1 mAh g^−1^, respectively. In comparison, all samples with the constructed LZCS passivation layer exhibited enhanced capacity and initial coulombic efficiency. Among them, the cell with LPSC-1.5%Z delivered an initial discharge capacity of 195.9 mAh g^−1^ and an initial coulombic efficiency of 85.3%, indicating that the passivation layer effectively suppressed parasitic interfacial reactions and improved the interfacial compatibility. Furthermore, a series of metal chloride precursors were employed under specific conditions to evaluate the applicability of this reconstruction strategy to different precursors. As summarized in Fig. S16, LPSC-1.5%ZrCl_4_ exhibited one of the most favorable electrochemical performances among all samples, indicating that the interfacial enhancement was strongly dependent on the nature of the metal halide precursor rather than arising from a universal effect of chloride addition alone.

The cycling performance of the different cells was evaluated at 0.5C. As shown in Fig. 4b and S17, the cell with LPSC-1.5%Z delivered an initial discharge capacity of 167.2 mAh g^−1^ and retained 95.4% of its capacity after 200 cycles, which was markedly higher than the retention values of 90.5% for the cell with LPSC and 91.9% for that with LPSC-S. Meanwhile, the cell with LPSC-1%Z, with a lower coating amount, exhibited a capacity retention of only 92.6%, indicating that insufficient coating was unable to effectively mitigate interfacial failure during long-term cycling. On the other hand, although the cell with LPSC-2%Z showed relatively high capacity retention, its discharge capacity decreased noticeably, which could be attributed to the decline in ionic conductivity caused by the excessive introduction of ZrCl_4_, thereby impairing the rate capability and limiting the overall battery performance. These results indicated that a moderate and uniform LZCS passivation layer effectively balanced interfacial ionic conductivity and interfacial shielding, thereby leading to the optimal cycling performance of the cell with LPSC-1.5%Z. Differential capacity (d*Q*/d*V*) analysis was further conducted to elucidate the reaction behavior of NCM811 in the all-solid-state cell. As shown in Fig. 4c, two cells exhibit the same phase transition behavior. The peaks located at around 3.0, 3.2–3.4, and 3.5–3.6 V can be assigned to the H1 + M, M + H2, and H2 + H3 processes, respectively.^40^ After 100 cycles, the characteristic peaks of the cell with LPSC were markedly attenuated, reflecting progressive interfacial deterioration during cycling. By comparison, the cell with LPSC-1.5%Z preserved these characteristic features with only slight variations in peak intensity and shape, indicating enhanced interfacial stability and better retention of structural reversibility over prolonged cycling.^46^

To evaluate the long-term cycling stability, Fig. 4d shows the evolution of discharge capacity and coulombic efficiency during cycling at 4.3 V and 1C. The cell with LPSC-1.5%Z delivered an initial discharge capacity of 147.1 mAh g^−1^ and retained 135.6 mAh g^−1^ after 1400 cycles, corresponding to a capacity retention of 91.4%, demonstrating excellent long-term cycling stability and sustained reaction reversibility. In contrast, the cell with LPSC delivered an initial discharge capacity of 140.8 mAh g^−1^, but retained only 76.1% of its capacity after 1400 cycles. Meanwhile, Fig. 4e shows the rate performance of the cells at different current densities. The cell with LPSC-1.5%Z delivered excellent rate capability, with discharge capacities of 192.2, 179.3, 175.3, 160.5, 139.7, 109.6, and 84.2 mAh g^−1^ at 0.1, 0.2, 0.3, 0.5, 1, 2, and 3C, respectively, whereas the cell with LPSC exhibited much more severe capacity decay at elevated rates, retaining only 57.2 mAh g^−1^ at 3C. The charge–discharge profiles at different rates further highlighted the distinct rate behaviors of LPSC and LPSC-1.5%Z (Fig. S18). The cell with LPSC-1.5%Z exhibited more stable voltage plateaus from 0.1 to 3C, with smaller voltage hysteresis and lower polarization. In contrast, the cell with LPSC showed a much more pronounced voltage drop at high current densities, suggesting aggravated polarization and less stable interfacial Li^+^ transport.

To further evaluate the stability of the reconstructed interphase, cycling tests were conducted at a cutoff voltage of 4.5 V and 1C. As shown in Fig. 4f, the cell with LPSC-1.5%Z retained a stable discharge capacity of 154.5 mAh g^−1^ after 600 cycles, corresponding to a capacity retention of 94.5%. In contrast, the cell with LPSC delivered only 131.6 mAh g^−1^ after 200 cycles, with a capacity retention of 87.1%. As summarized in Fig. 4g, comparison with previous studies showed that the cell with LPSC-1.5%Z achieved advanced performance in sulfide-based ASSLBs, exhibiting outstanding high-voltage tolerance and excellent cycling stability.^47–53^

Finally, to determine whether the performance improvement originated from the direct effect of ZrCl_4_ itself, a physically mixed sample (LPSC-Z-PM) was evaluated (Fig. S19). It delivered an ICE of 82.0% and an initial discharge capacity of 183.5 mAh g^−1^, and its discharge capacity decreased to 136.4 mAh g^−1^ after 100 cycles at 1C, all of which were lower than those of LPSC-1.5%Z. These results demonstrated that simple ZrCl_4_ addition was insufficient and that effective passivation required a controlled reaction between ZrCl_4_ and the surface S species to construct the LZCS passivation layer. The resulting layer chemically shielded the highly reactive sulfur sites, thereby enhancing interfacial stability. Its favorable compatibility with the LPSC surface further enabled sustained protection during long-term cycling and high-voltage operation.

**Fig. 5:**
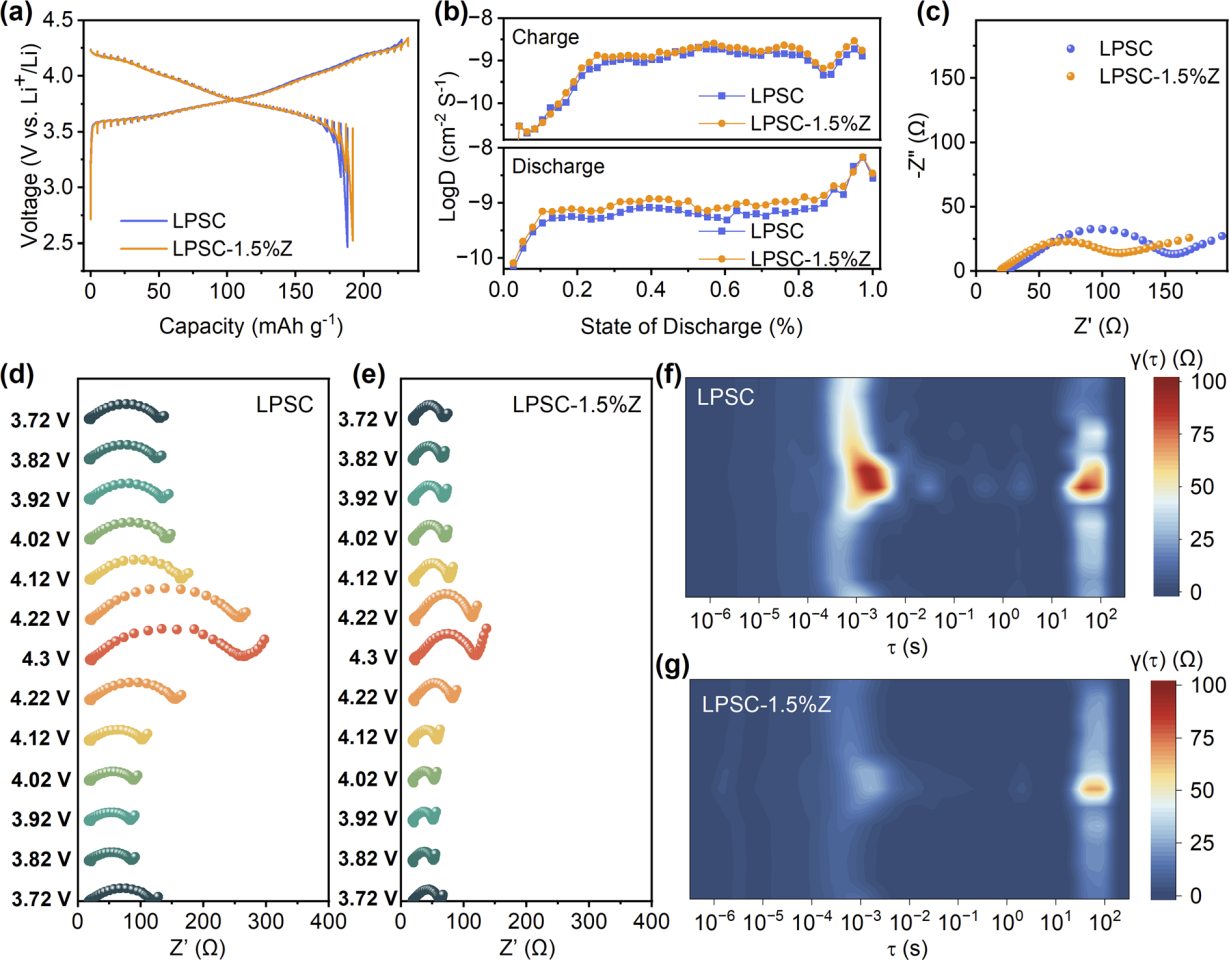
Dynamic behavior of LPSC and LPSC-1.5%Z systems. (a) GITT curves for the cells with LPSC and LPSC-1.5%Z after activation, (b) Li^+^ diffusion coefficient, (c) Nyquist plots after 100 cycles, (d and e) *in situ* Nyquist spectra during the second cycle, and (f and g) DRT heatmaps from EIS data.

To gain deeper insight into the relationship between interfacial kinetics and interphase reconstruction, the Li^+^ transport behavior during charge–discharge cycling was first analyzed by the galvanostatic intermittent titration technique (GITT). Fig. 5a shows the voltage–capacity profiles during charge and discharge after one activation cycle, reflecting the polarization response of the cells under intermittent current perturbation. The apparent Li^+^ diffusion coefficients derived from the GITT data are presented in Fig. 5b as a function of the state of charge (SOC). Compared with pristine LPSC, the cell with LPSC-1.5%Z exhibited slightly higher and more stable apparent Li^+^ diffusion coefficients throughout charge and discharge, especially during discharge. This improvement arose from the LZCS passivation layer, which suppressed interfacial by-product formation while preserving the relatively high ionic conductivity of the modified electrolyte, thereby enhancing Li^+^ transport during cycling.

To further evaluate the interfacial evolution during long-term cycling, electrochemical impedance spectroscopy (EIS) was performed on the cells after 100 cycles. As shown in Fig. 5c, the impedance of the cell with LPSC-1.5%Z was significantly lower than that of the cell with LPSC. The Nyquist plots were fitted using an equivalent circuit model (Fig. S20 and S21), which showed that the cells exhibited similar solid electrolyte resistance (*R*_s_) and grain-boundary resistance (*R*_gb_) after cycling. In contrast, the main difference was reflected in the charge-transfer resistance (*R*_ct_), which represented the resistance associated with the electrochemical reaction involving ions and electrons at the electrode/electrolyte interface.^20^ The *R*_ct_ decreased from 91.0 Ω for the cell with pristine LPSC to 38.3 Ω for the cell with LPSC-1.5%Z. This reduction could be attributed to the ability of the LZCS interfacial layer to suppress the formation of resistive interfacial by-products by passivating the highly reactive S sites, while its good chemical compatibility with LPSC rendered the interphase less susceptible to rapid failure during cycling. As a result, the *in situ* reconstructed LZCS interphase effectively lowered the charge-transfer resistance at the interface.

To further probe the dynamic evolution of interfacial impedance during cell operation, *in situ* EIS measurements were conducted. Fig. S22 shows the selected charge–discharge states and sampling points for the *in situ* EIS measurements, while Fig. 5d and e present the corresponding *in situ* Nyquist spectra collected at different potentials. For unmodified LPSC, the overall resistance variation reached about 120 Ω over the charge–discharge process, revealing severely hindered Li^+^ transport across the interface. In contrast, the cell with LPSC-1.5%Z showed a much smaller resistance variation of only about 50 Ω, and the spectra exhibited much smaller changes during cycling, indicating that the LZCS interfacial layer effectively suppressed impedance buildup. To gain deeper insight into the interfacial dynamics, the *in situ* EIS results were further converted into distribution of relaxation time (DRT) plots (Fig. S23 and S24) and visualized as heatmaps to highlight the impedance contributions at different time scales as a function of potential. According to previous studies on solid-state battery systems, different relaxation-time intervals could be assigned to distinct electrochemical processes. As shown in Fig. 5f and g, the cells with LPSC and LPSC-1.5%Z exhibited substantially different impedance evolution behaviors over different time scales. In the high-frequency region (10^−6^–10^−5^ s), the impedance was generally associated with the grain-boundary resistance (*R*_gb_), as well as the resistance of the electrolyte interphase (*R*_SEI_).^54^ During cycling, the cell with LPSC-1.5%Z exhibited slightly higher *R*_gb_ and *R*_SEI_ values than that with LPSC, which was associated with the slight decrease in intrinsic ionic conductivity induced by the introduction of the interfacial layer. The most pronounced variation was observed in the mid-to-high-frequency region (10^−4^–10^−2^ s), where the impedance predominantly reflected Li^+^ migration through the cathode/electrolyte interphase (*R*_CEI_).^40^ Upon charging to 3.68 V, the *R*_CEI_ of the cell with LPSC increased markedly, indicating severe interfacial degradation caused by the direct contact between LPSC and NCM811. This behavior was attributed to interfacial side reactions and the formation of poorly conductive decomposition products, which sharply increased the interfacial impedance. In contrast, the cell with LPSC-1.5%Z showed only a slight increase in *R*_CEI_, indicating that the uniformly distributed LZCS passivation layer mitigated the formation of poorly ionically conductive by-products by preventing direct contact between reactive S species and the cathode, while helping maintain more stable interfacial contact between the cathode and electrolyte. In the low-frequency region (10^0^–10^2^ s), the impedance was mainly associated with diffusion processes and long-range Li^+^ transport within the cathode (*R*_diffusion_).^46^ The cell with LPSC exhibited a pronounced increase in this impedance contribution, indicating unfavorable structural evolution at the cathode surface during cycling, likely associated with microcrack formation in NCM811 under highly charged conditions. In contrast, the *R*_diffusion_ of the cell with LPSC-1.5%Z showed a much smaller increase, suggesting that the LZCS interphase reduced interfacial side reactions and stress evolution, while its amorphous nature likely provided additional buffering capability, thereby alleviating impedance buildup during cycling. Taken together, these results highlighted the dual role of the LZCS passivation layer in improving interfacial Li^+^ transport and alleviating interfacial stress.

**Fig. 6:**
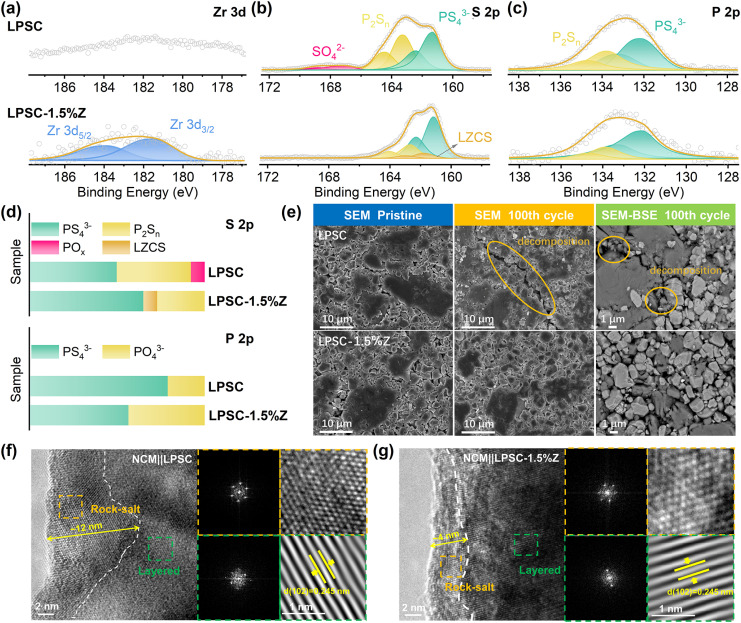
Characterization of composite cathodes in LPSC and LPSC-1.5%Z systems: (a) Zr 3d, (b) S 2p, and (c) P 2p XPS spectra of composite cathodes after 100 cycles. (d) The percentages of various groups identified from the S 2p and P 2p spectra. (e) SEM images of composite cathodes before and after 100 cycles, including top-view SEM-BSE images. HRTEM images and corresponding FFT patterns of NCM811 in (f) NCM811‖LPSC and (g) NCM811‖LPSC-1.5%Z after 100 cycles.

X-ray photoelectron spectroscopy (XPS) was performed on the composite cathodes before and after 100 cycles to examine the evolution of the interfacial chemical composition during cycling. As shown in Fig. 6a–c, the detected Zr signal in LPSC-1.5%Z was assigned to the introduced LZCS-related species. In the S 2p spectrum of LPSC, in addition to the intrinsic PS_4_^3−^ signals, the appearance of P_2_S_*n*_ peaks at 163.4 and 164.5 eV, together with SO_4_^2−^ signals at 167.3 and 168.9 eV, indicated substantial decomposition and oxidation of sulfur-containing species.^40,53^ In contrast, the S 2p spectrum of LPSC-1.5%Z still retained clear LZCS-related features, indicating that the passivation layer remained present after cycling. The significantly suppressed P_2_S_*n*_ signal suggested that the LZCS layer effectively inhibited sulfide decomposition, while the nearly undetectable sulfur oxidation products indicated that the reaction between S species and cathode oxygen was substantially suppressed.^37^ Similarly, the P 2p spectrum of LPSC showed distinct P_2_S_*n*_ related signals at 133.8 and 134.8 eV, whereas LPSC-1.5%Z exhibited markedly weakened P_2_S_*n*_ peaks,^40^ further suggesting that the LZCS layer formed through the reaction between Zr and S acted as an effective shielding interphase, which not only isolated direct physical contact but also reduced the reactivity of S species. The relative proportions of these species were further quantified, as summarized in Fig. 6d. Compared with LPSC, the LPSC-1.5%Z composite cathodes retained a higher proportion of the bulk-phase species after cycling and showed significantly reduced formation of the P_2_S_*n*_ and SO_4_^2−^ phase. These results demonstrated that the LZCS layer effectively mitigated the oxidation and decomposition of the sulfide electrolyte at the cathode interface, thereby enhancing interfacial chemical stability.

Scanning electron microscopy (SEM) was further employed for characterization of the cathode/electrolyte interface, as shown in Fig. 6e. Before cycling, the composite cathodes with LPSC and LPSC-1.5%Z exhibited intimate contact between the cathode active material and the sulfide electrolyte, indicating good initial physical contact within the composite cathode. After 100 cycles, the composite cathode with LPSC showed pronounced cracks and localized regions of interfacial contact failure, accompanied by morphological features indicative of electrolyte decomposition. In contrast, the composite cathode with LPSC-1.5%Z retained a dense and uniform morphology after cycling, with no obvious pores or severe electrolyte decomposition. These results suggested that, in the uncoated system, the continuous accumulation of interfacial by-products induced local volume changes and stress concentration, thereby promoting pore formation, crack propagation, and interfacial contact failure, which further restricted interfacial transport. In contrast, the LZCS layer reduced the formation of interfacial by-products, thereby alleviating the local structural degradation caused by their inhomogeneous accumulation. Meanwhile, its amorphous nature likely provided a buffering effect against the cathode volume changes during charge–discharge cycling, thereby suppressing pore and crack formation.

Furthermore, high-resolution transmission electron microscopy (HRTEM) was employed to directly compare the surface structural evolution of cycled NCM811 particles in the LPSC and LPSC-1.5%Z systems. As shown in Fig. 6f and g, for the NCM811 cathode in the LPSC system after 100 cycles, the lattice spacing measured in the particle interior was 0.245 nm, corresponding to the (102) plane, indicating that the layered structure in the bulk region was largely preserved. In contrast, Fast Fourier Transform (FFT) analysis of the surface region revealed diffraction features characteristic of the rock-salt phase, indicating that a localized layered to rock-salt transformation had occurred on the particle surface during cycling. In the uncoated LPSC system, the rock-salt phase on the NCM811 surface was unevenly distributed, with a maximum thickness of approximately 12 nm, demonstrating severe surface structural degradation. By contrast, in the LPSC-1.5%Z system, the rock-salt reconstruction was confined to the outermost surface region, exhibiting a much more uniform distribution and a thickness of only ∼4 nm. This result indicated that the LZCS interfacial layer effectively suppressed the surface layered to rock-salt transition and significantly retarded its propagation into the particle interior, demonstrating that the electrolyte-derived passivation layer also played a beneficial role in mitigating the surface structural degradation of the cathode.

## Conclusions

In summary, this work proposes a coordination-driven interfacial reconstruction strategy to construct a uniformly distributed Li–Zr–Cl–S (LZCS) passivation layer *in situ* on the LPSC surface by exploiting the volatility and strong Lewis acidity of ZrCl_4_. The reconstructed interphase forms through the reaction between Zr and S-related species, simultaneously providing chemical passivation of highly reactive S sites, thereby suppressing the reaction between the sulfide electrolyte and the Ni-rich cathode. Moreover, due to its excellent chemical compatibility with the LPSC host, the interphase resists rapid degradation during long-term cycling, maintaining sustained interfacial protection. Benefiting from these synergistic effects, the ASSLBs incorporating the interface-modified LPSC electrolyte and NCM811 cathode deliver outstanding cycling stability, with 91.4% capacity retention after 1400 cycles at 1C. Notably, the cells also demonstrate outstanding cycling stability at 4.5 V, with a capacity retention of 94.5% after 600 cycles. This chemical passivation strategy, which leverages S-site deactivation and halide interphase formation, establishes a general design principle for sulfide electrolyte modification and high-performance solid-state batteries.

## Author contributions

Wenrui Hu: experimental work, writing – original draft, writing – review and editing; Qingkun Zhu: DRT and EIS data analysis; Fenghua Zheng: some experimental data analysis; Xinyou He: some experimental data analysis; Heng Wen: some experimental data analysis; Zhiming Xiao: TEM data analysis; Wei Xu: TEM data analysis; Fenghua Ding: some battery assembly work; Xinghui Liang: supervision, writing – review and editing; Lei Ming: writing – review and editing, funding acquisition; Baishan Chen: writing – review and editing, funding acquisition; Changsheng An: writing – review and editing, funding acquisition; Zhang Lin: some experimental data analysis; Xing Ou: supervision, writing – review and editing, funding acquisition. All the authors discussed the experimental results.

## Conflicts of interest

There are no conflicts to declare.

## Supplementary Material

SC-OLF-D6SC03904A-s001

## Data Availability

The data supporting the findings of this study are available within the article and its supplementary information (SI). Supplementary information: experimental details, supplementary figures, electrochemical analysis, and characterization data. See DOI: https://doi.org/10.1039/d6sc03904a.
