# Supplementary material for: Surface reconstruction of metal halides for S-site shielding toward long-cycle all-solid-state lithium batteries
Source: Chem Sci. 2026 Jun 30;17(33):15801–12. doi: 10.1039/d6sc03904a (PMC13343528; doi:10.1039/d6sc03904a)
Supplement: Supplementary file 1 [file SC-017-D6SC03904A-s001.pdf]

Supplementary Information (SI) for Chemical Science.
This journal is © The Royal Society of Chemistry 2026

Electronic Supporting Information

Surface Reconstruction of Metal Halides for S-Site Shielding toward Long-Cycle All-Solid-State Lithium Batteries

Wenrui Hu,[a] Qingkun Zhu,[a] Fenghua Zheng,[b] Xinyou He,[a] Heng Wen,[a] Zhiming Xiao,[a] Wei Xu,[a,c] Fenghua Ding,[a] Xinghui Liang,[a,*] Lei Ming,[a] Baishan Chen,[d,*] Changsheng An,[e,*] Zhang Lin,[a] Xing Ou[a,*]

[a] Engineering Research Center of the Ministry of Education for Advanced Battery Materials, School of Metallurgy and Environment, Central South University, Changsha, 410083, P.R. China

[b] Guangxi Key Laboratory of Low Carbon Energy Materials, School of Chemistry and Pharmaceutical Sciences, Guangxi Normal University, Guilin, 541004, PR China

[c] Guangxi Huayou Lithium Industry Co., Ltd. Yulin, 537624, China

[d] Institute for Advanced Study, Central South University, Changsha 410083, China

[e] School of Materials and Environmental Engineering, Changsha University, Changsha 410022, P.R. China

Experimental section

Materials preparation

Li_2S, P_2S_5 and LiCl (all purchased from Aladdin) were used as starting materials for the synthesis of Li_6PS_5Cl (denoted as LPSC). The reagents were weighed according to the stoichiometric ratio and thoroughly mixed by ball milling in a ZrO_2 jar for 12 h under an Ar atmosphere. The obtained precursor powder was then transferred to a muffle furnace under flowing Ar and annealed at 550 °C for 8 h. After cooling to room temperature, the product was further ground using an agate mortar to obtain the final LPSC powder. Unless otherwise noted, all procedures were carried out under an Ar atmosphere. For the synthesis of LPSC-Z, $ZrCl_4$ was first ground in a mortar into powder. Subsequently, LPSC and $ZrCl_4$ were mixed at a designed mass ratio and ground sufficiently to ensure uniformity. The resulting mixture was transferred into a sealed glass container and heat-treated in a muffle furnace at 400 °C for 2 h under an Ar atmosphere. After cooling to room temperature, the product was collected and ground in a mortar to obtain the powder. Unless otherwise noted, all procedures were conducted under an Ar atmosphere.

To Prepare other modified LPSC, $AlCl_3$, $GaCl_3$, VCl_3, $NbCl_5$, and $TiCl_4$ were used as precursors to prepare the corresponding modified LPSC electrolytes. For the solid metal chlorides, the treatment temperatures were selected according to their respective volatilization or sublimation characteristics. Owing to its liquid state, the $TiCl_4$ modified sample was prepared by directly immersing LPSC in liquid $TiCl_4$ under an inert atmosphere.

Solid-State Cell Assembly

All-solid-state cells were fabricated inside an Ar-filled glovebox. To prepare the composite cathode, single-crystal NCM811 (scNCM811) and solid electrolyte powder were mixed at a mass ratio of 7:3 and manually ground in an agate mortar for 20 min. During cell assembly, 80 mg of solid electrolyte powder was first placed into a 10 mm polyetheretherketone (PEEK) mold and uniaxially compressed at 340 MPa for 1 min to obtain a compact electrolyte layer. Then, 10 mg of the prepared composite cathode powder was uniformly

distributed onto the electrolyte layer and pressed again at 340 MPa for 5 min. On the other side of the pellet, an In foil with a diameter of 10 mm and a Li foil with a diameter of 4 mm were successively attached. The assembled cells were maintained under 70 MPa for 6 h, followed by electrochemical measurements under a constant stack pressure of 50 MPa.

Electrochemical Characterization

Ionic conductivity: A symmetric cell composed of stainless steel||electrolyte||stainless steel was assembled for electrochemical impedance spectroscopy (EIS) analysis. The measurements were performed over a frequency window ranging from 1 MHz to 1 Hz at 30 °C. The ionic conductivity (σ) was subsequently determined using Equation (1):

$$\sigma = \frac{L}{RS} \quad (1)$$

where L is the thickness of the electrolyte (cm), R is the total impedance (Ω), and S is the area of the stainless steel electrode (diameter: 10 mm).[1, 2]

Electronic conductivity: A stainless steel||electrolyte||stainless steel ion-blocking configuration was constructed to evaluate the electronic conductivity using direct-current (DC) polarization. In this setup, the solid electrolyte pellet was positioned between two stainless steel blocking electrodes, and a constant DC bias was imposed across the cell. The resulting polarization current was monitored continuously over 3 h until a stable plateau was achieved. The electronic conductivity (σe) was subsequently derived from this steady-state current according to Equation (2):

$$\sigma_e = \frac{LI}{SE} \quad (2)$$

where σ_e is the electronic conductivity, usually expressed in mS cm^{-1}, L is the thickness of the solid electrolyte pellet, S is the area of the stainless steel electrode (diameter: 10 mm), I is the steady-state current obtained from the DC polarization curve, and E is the applied polarization voltage (E=0.1 V).[3]

Lithium-ion diffusion coefficient：The lithium-ion diffusion coefficient (D_{Li^+}) was evaluated using the

galvanostatic intermittent titration technique (GITT). Full cells were assembled and tested at 30 °C under a current rate of 0.1 C. During the measurements, repeated sequences of current pulses followed by relaxation periods were applied, while the corresponding voltage evolution under both polarization and relaxation states was continuously recorded. The diffusion coefficient was then calculated according to Equation (3):

$$D_{Li^+} = \frac{4}{\pi}\left(\frac{I_0 V_m}{SFz_i}\right)^2 \left(\frac{dE/dx}{dE/dx^{\frac{1}{2}}}\right)^2 \tag{3}$$

where I_0 is the applied current (A), V_m is the molar volume ($cm^3\ mol^{-1}$), S is the contact area between the electrode and the electrolyte (cm^2), F is the Faraday constant (96485 C mol^{-1}), z_i is the charge number of the transferring ion, and x represents the state of charge or discharge progress.[1]

Galvanostatic charge-discharge tests of ASSLBs were performed using a LAND battery test system (CT2001A) within voltage windows of 2.18–3.68 V and 2.18–3.88 V versus Li–In, corresponding to 2.8–4.3 V and 2.8–4.5 V versus Li^+/Li, respectively.[4, 5] The first three cycles were carried out as an activation process at 0.1 C (1 C = 200 mA h g^{-1}), followed by long-term cycling at designated rates. EIS measurements were also conducted for ASSLBs before and after cycling in the frequency range from 1 MHz to 1 mHz.

Characterizations

The crystalline structures were characterized by X-ray diffraction (XRD) on a powder X-ray diffractometer with Cu Kα radiation (λ = 1.54059 Å, 40 mA, 45 kV; Malvern Panalytical B.V., Netherlands). A 10 μm polyimide film was applied as a protective layer to avoid corrosion caused by moisture exposure. Raman spectra were collected to provide additional information on the local structural environments. The microstructural features were investigated using scanning electron microscopy (SEM, JEOL JSM-7800F), and the corresponding elemental mapping was obtained by energy-dispersive spectroscopy (EDS). The chemical states of the elements were determined by X-ray photoelectron spectroscopy (XPS, Thermo Scientific ESCALAB 250Xi). Before XPS analysis, the samples were placed in a customized vacuum transfer chamber and rapidly transferred into the analysis chamber to prevent contact with air. High-magnification morphology,

lattice fringes, and diffraction patterns were examined by transmission electron microscopy (TEM, JEOL JEM-2100F). The cathode and electrolyte samples were analyzed at accelerating voltages of 200 and 80 kV, respectively. Surface chemical compositions were further investigated by time-of-flight secondary ion mass spectrometry (TOF-SIMS, IONTOF GmbH 5-100).

First-principles calculations.

All first-principles calculations were performed within the framework of density functional theory (DFT) using the Vienna Ab initio Simulation Package (VASP). The interactions between ionic cores and valence electrons were described by the projector augmented-wave (PAW) method. The exchange–correlation potential was treated using the Perdew–Burke–Ernzerhof (PBE) functional within the generalized gradient approximation (GGA). To account for long-range van der Waals interactions, the DFT-D3 dispersion correction was included in all calculations. The plane-wave kinetic energy cutoff was set to 650 eV. The electronic self-consistent-field convergence criterion was set to 1×10^{-5} eV, and all structures were fully relaxed until the residual forces on each atom were less than 0.01 eV $Å^{-1}$. A Γ-centered 3 × 3 × 1 k-point mesh was used for structural optimization. A vacuum layer larger than 15 Å was introduced along the direction perpendicular to the surface to avoid interactions between periodic images. Differential charge density analysis was performed to evaluate the interfacial charge redistribution after adsorption. The charge density difference was calculated as

$$\Delta\rho = \rho_{total} - \rho_{surface} - \rho_{adsorbate}$$

, where ρ_{total}, $\rho_{surface}$, and $\rho_{adsorbate}$ represent the charge densities of the optimized combined system, the isolated substrate, and the isolated adsorbate, respectively. The isolated components were calculated using the same atomic positions and computational settings as those in the optimized combined system.

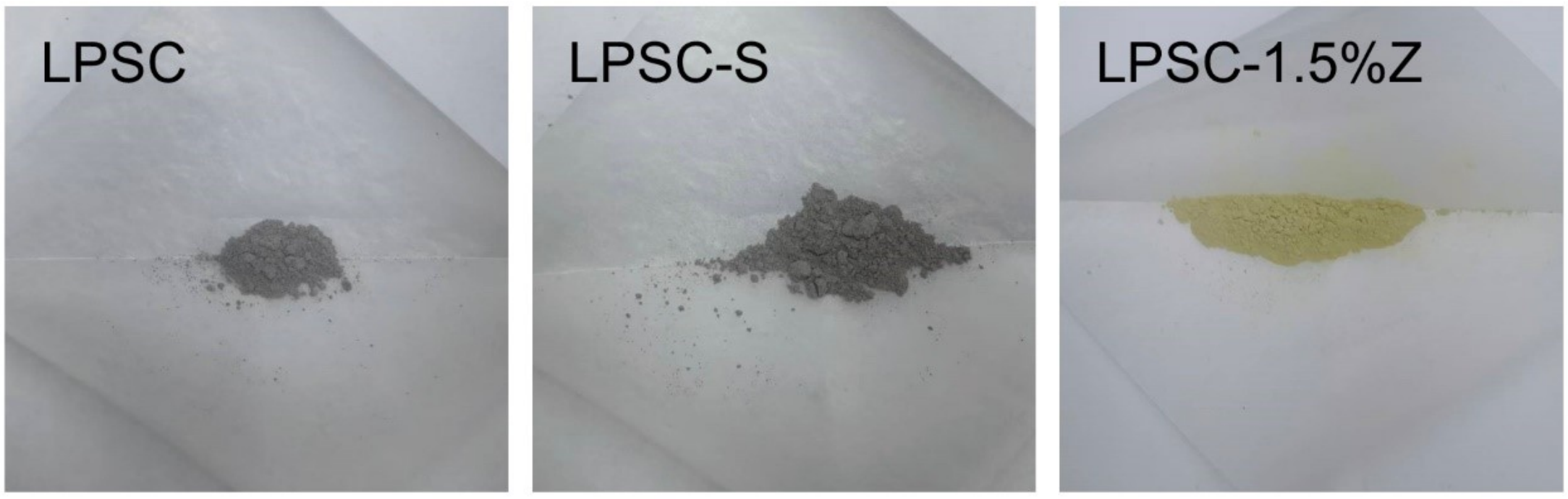

Fig. S1. Optical images of the samples

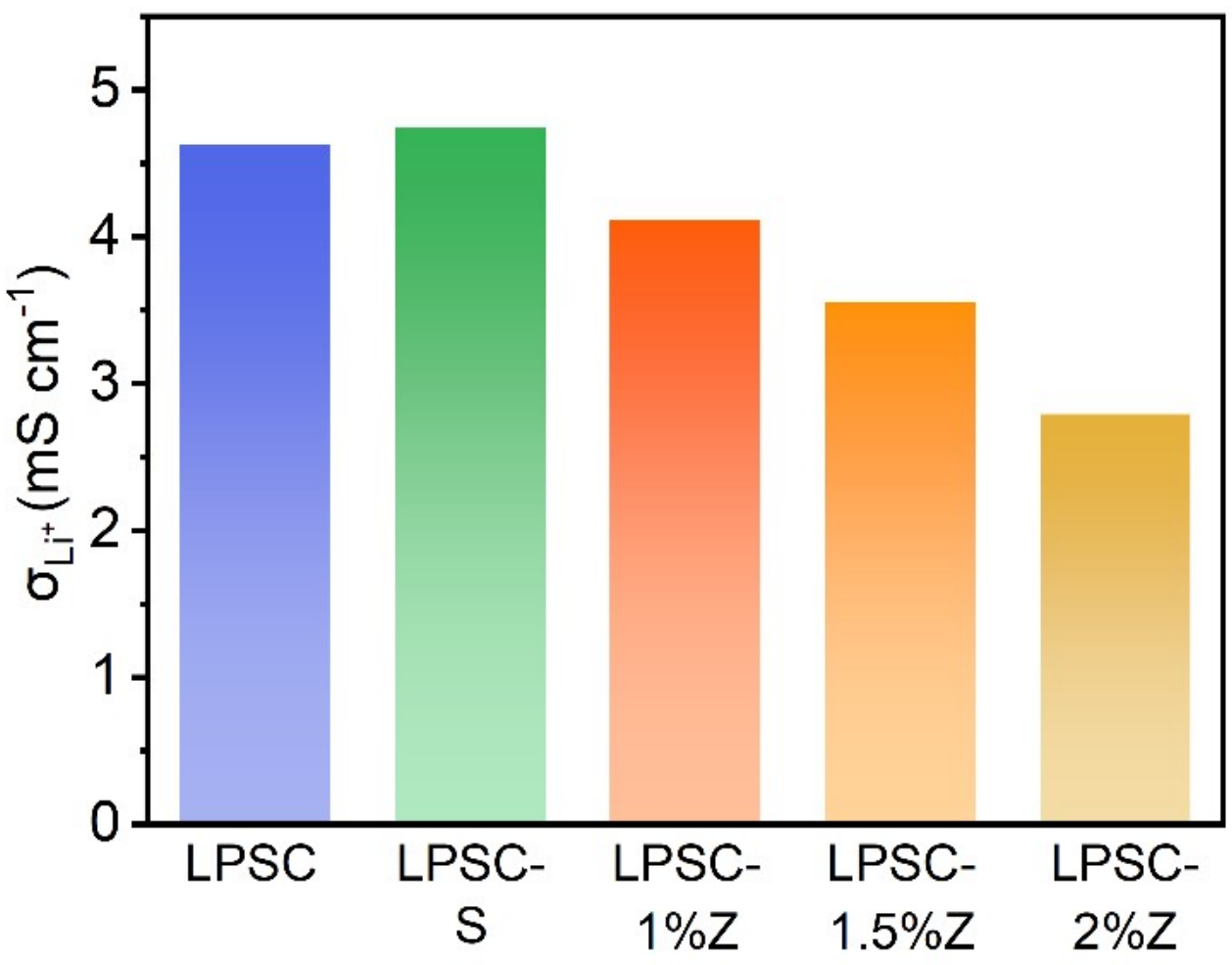

Fig. S2. Ionic conductivity of pristine LPSC, LPSC-S, and LPSC with different $ZrCl_4$ coating amounts

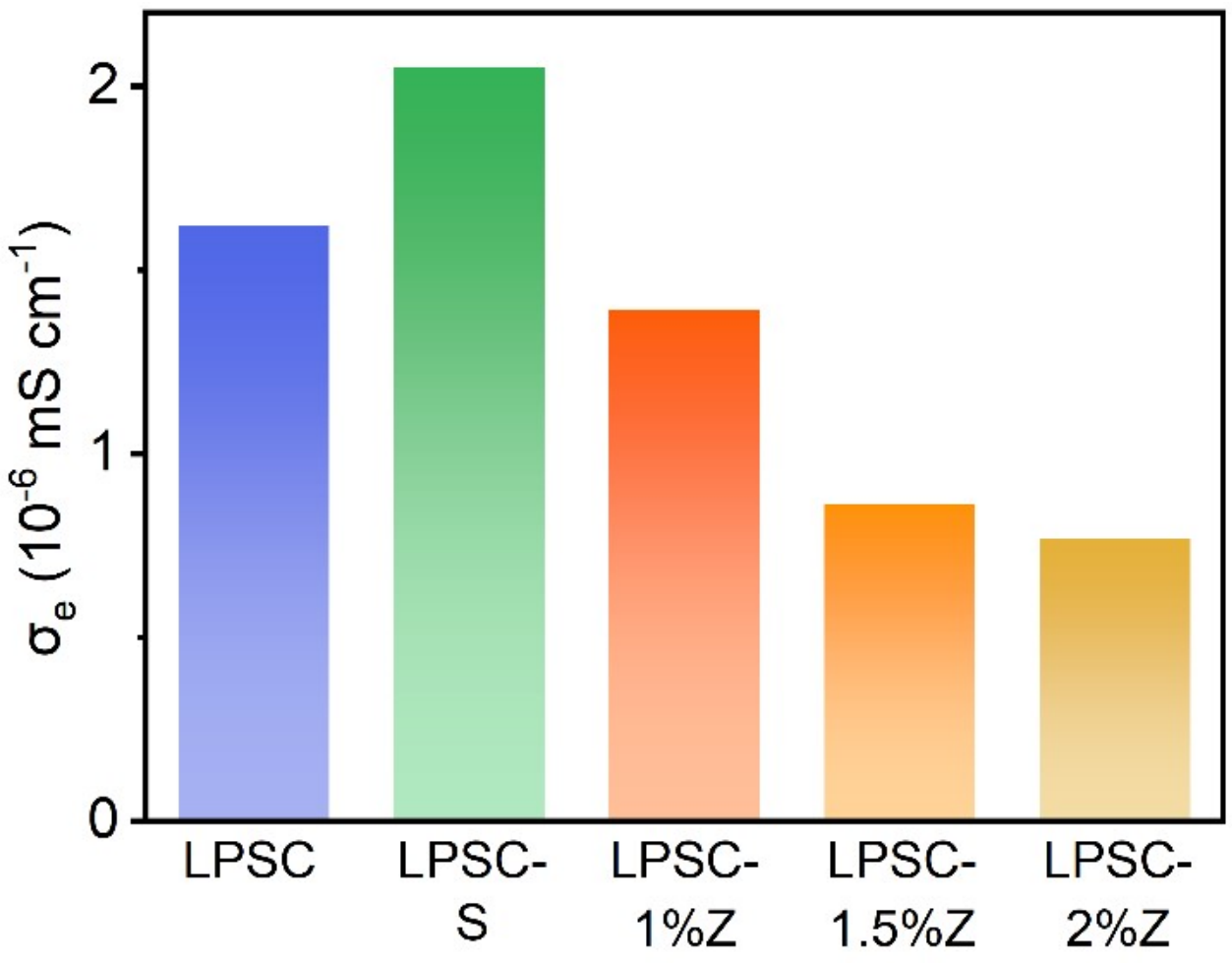

Fig. S3. Electronic conductivity of pristine LPSC, LPSC-S and LPSC with different $ZrCl_4$ coating amounts

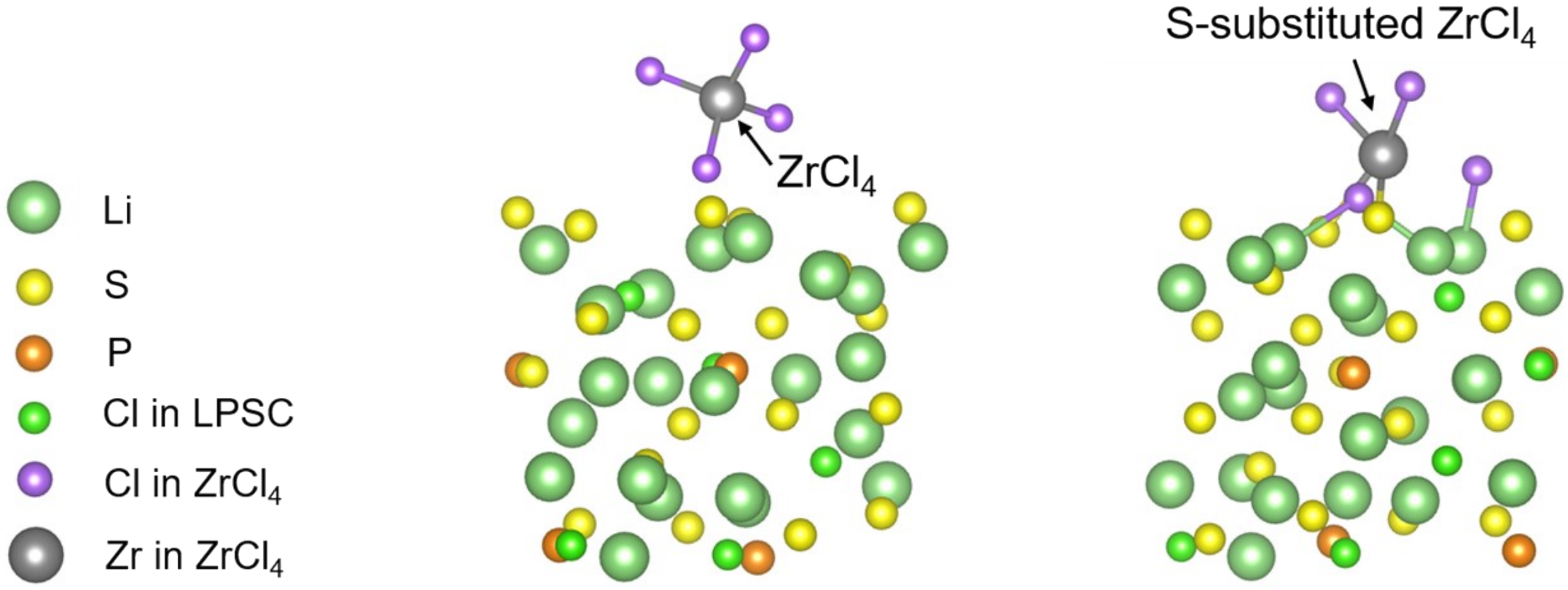

Fig S4. DFT simulation of the reaction between $ZrCl_4$ and LPSC

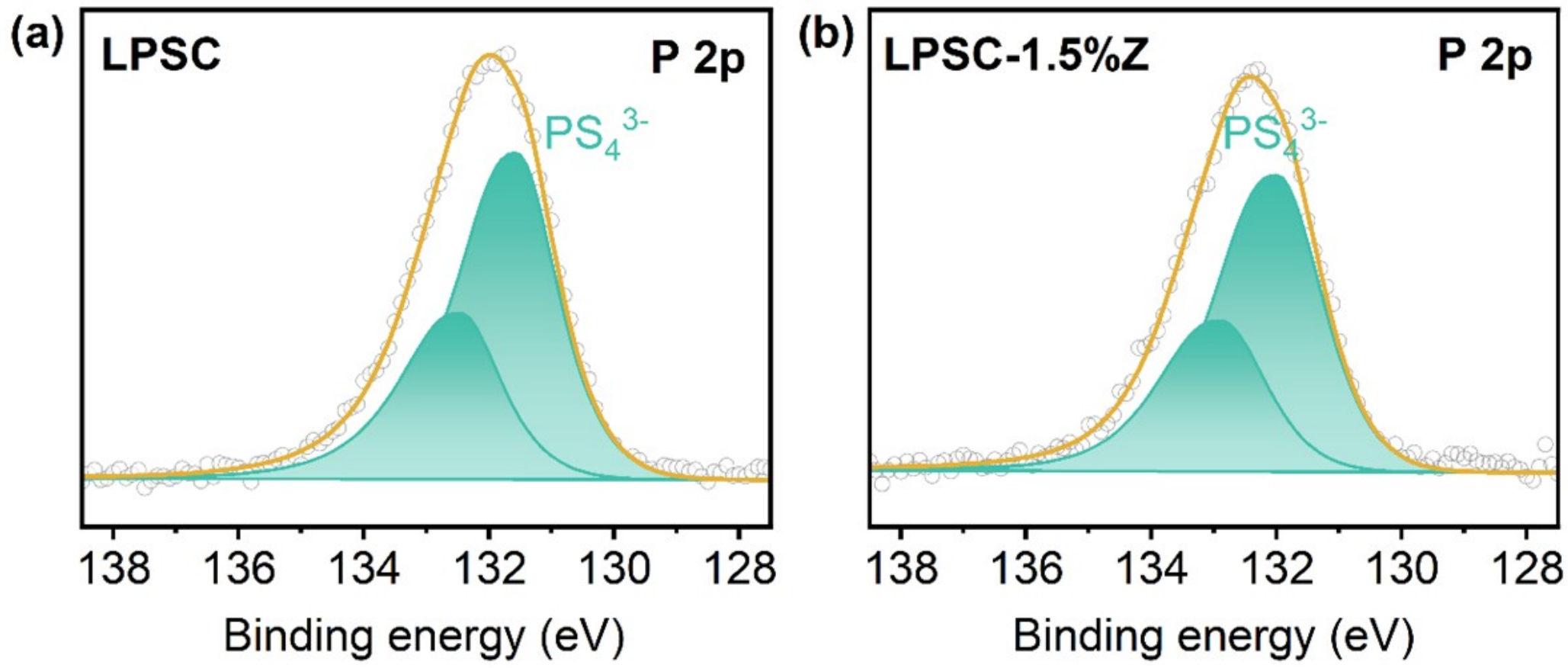

Fig. S5. P 2p XPS spectra of (a) LPSC and (b) LPSC-1.5%Z systems

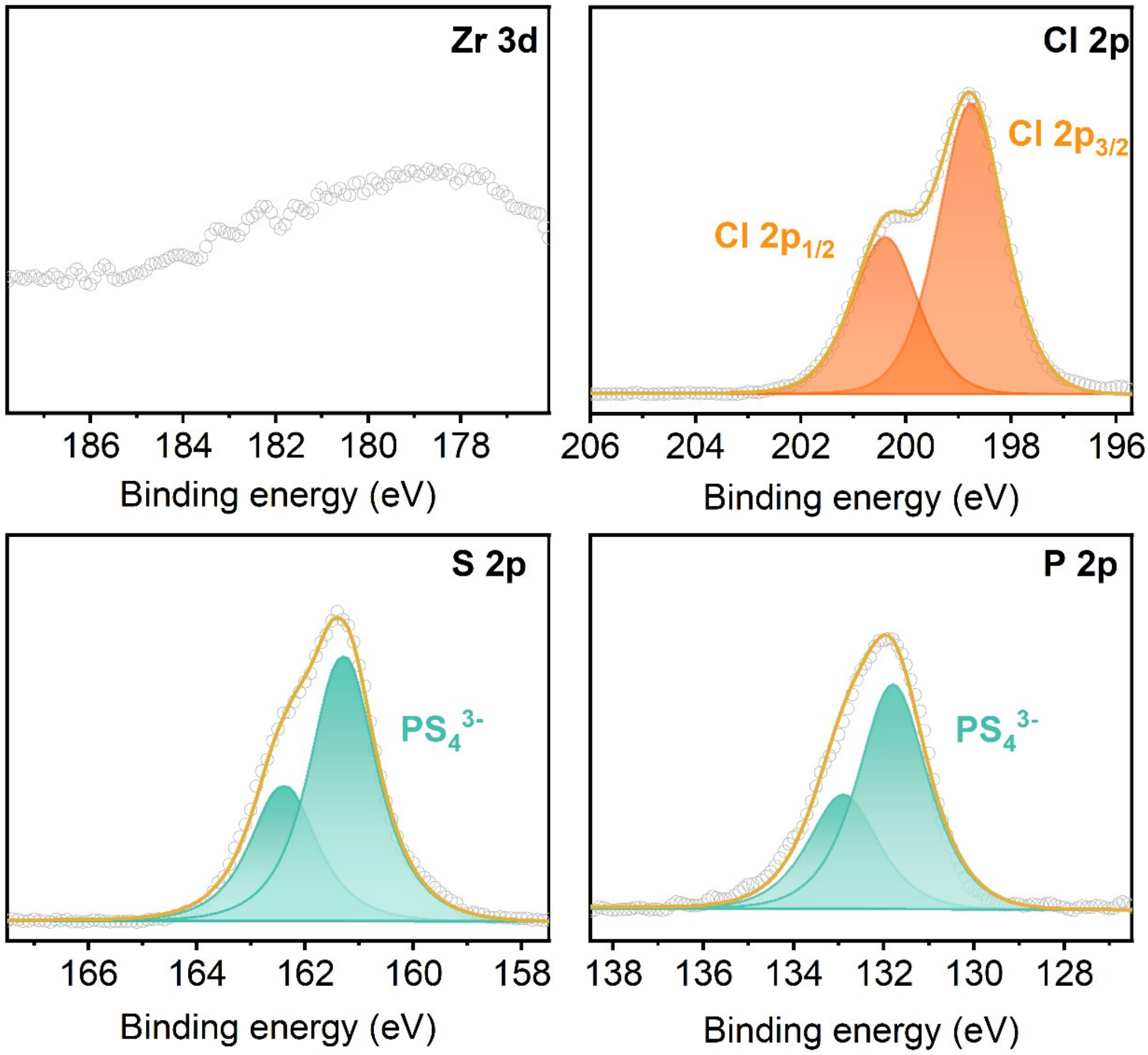

Fig. S6.XPS spectra of LPSC-S

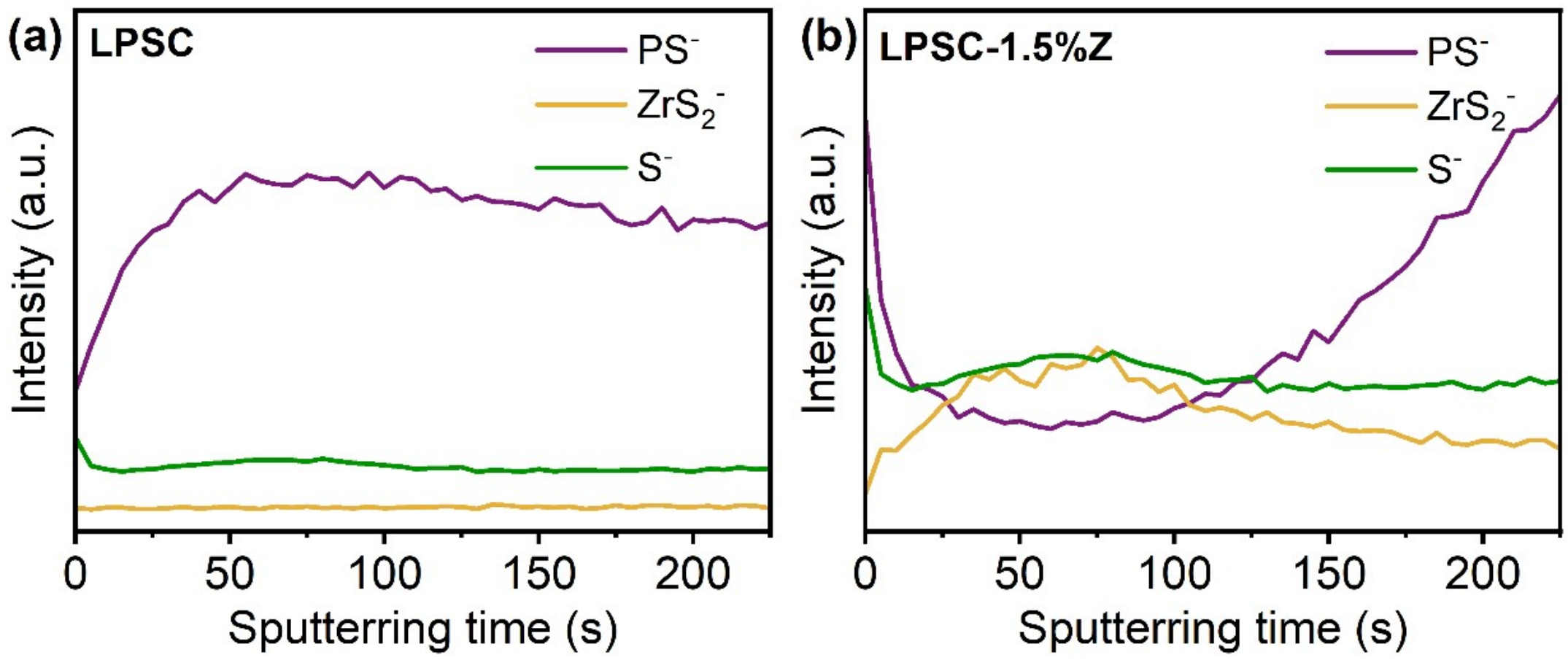

Fig. S7. TOF-SIMS depth profiles of (a) LPSC and (b) LPSC-1.5%Z obtained during sputtering, showing the ionic distributions of PS^-, ZrS_2^- and S^- secondary-ion fragments as a function of sputtering time.

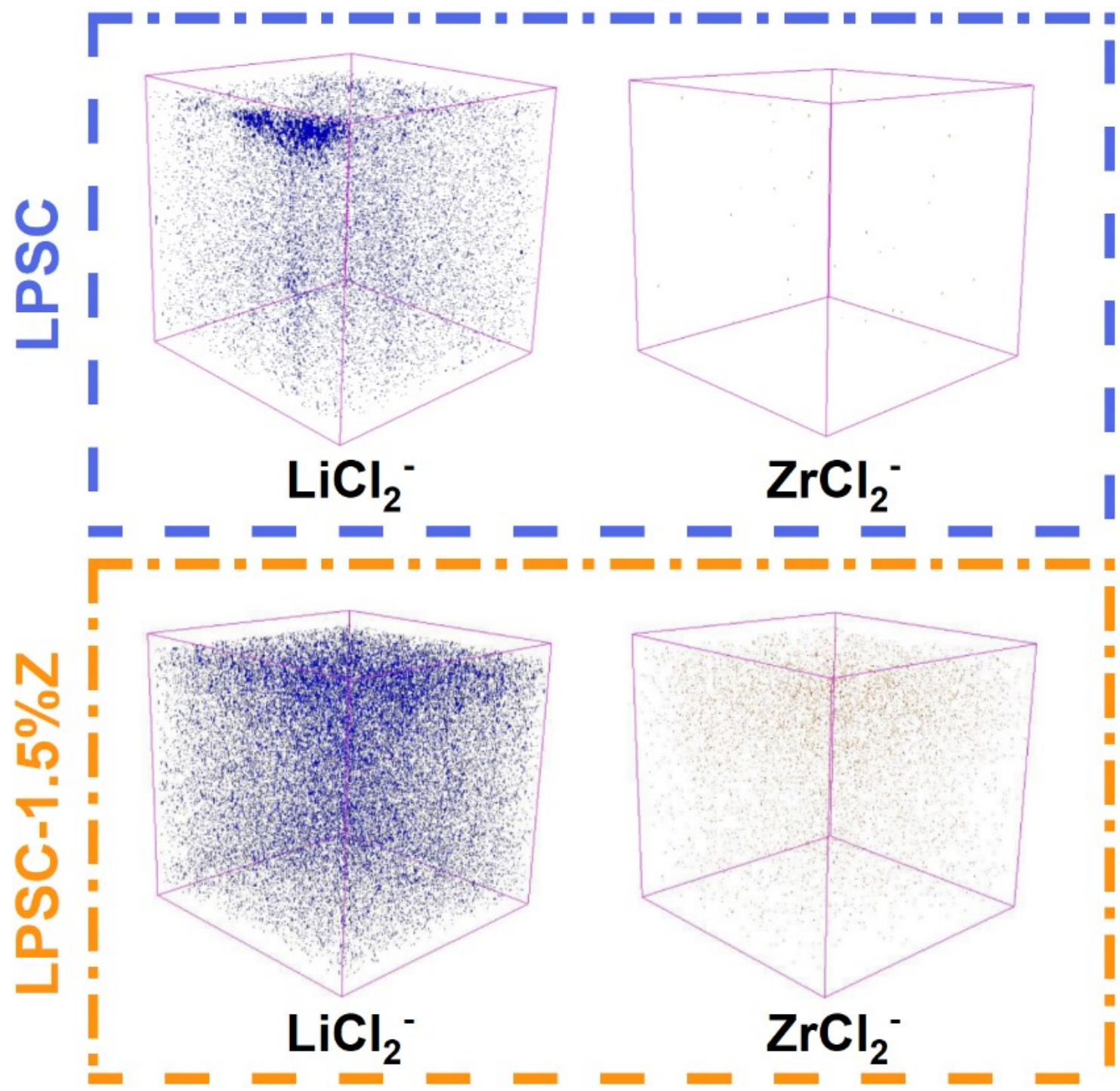

Fig. S8. TOF-SIMS fragments of $LiCl_2^-$ and $ZrCl_2^-$ in LPSC and LPSC-1.5%Z

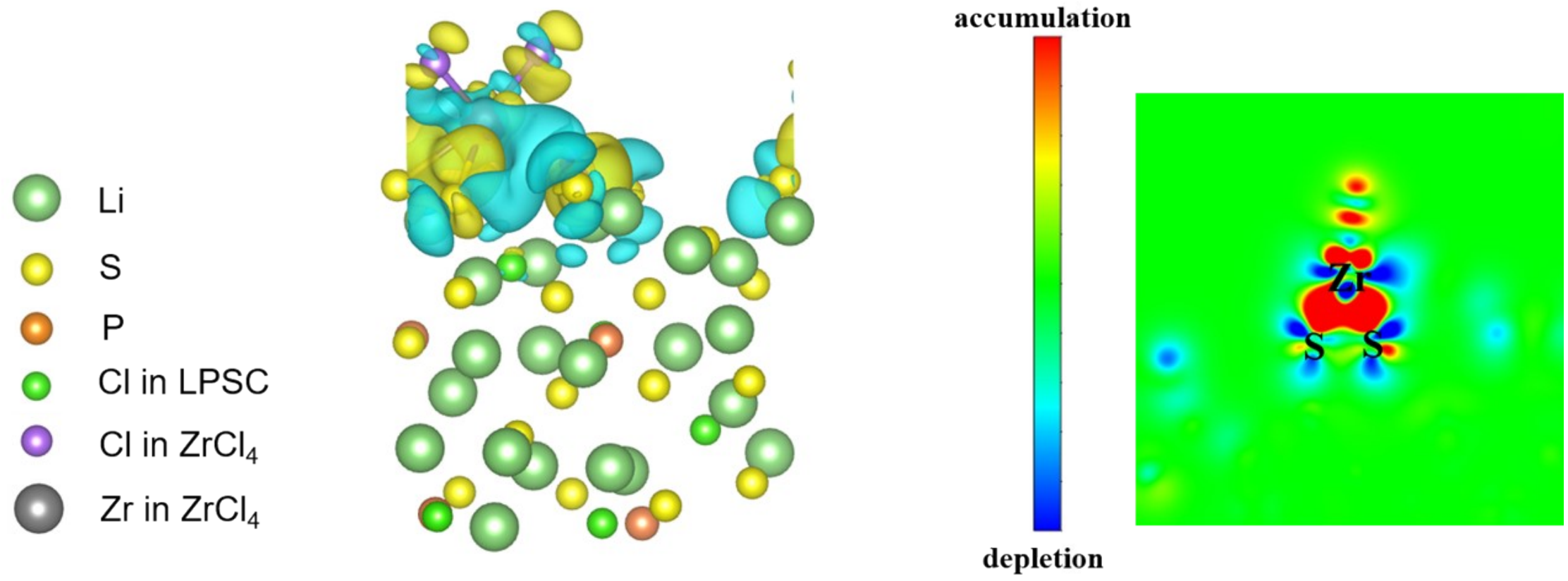

Fig. S9. Charge density difference of the passivation layer in LPSC-1.5%Z.

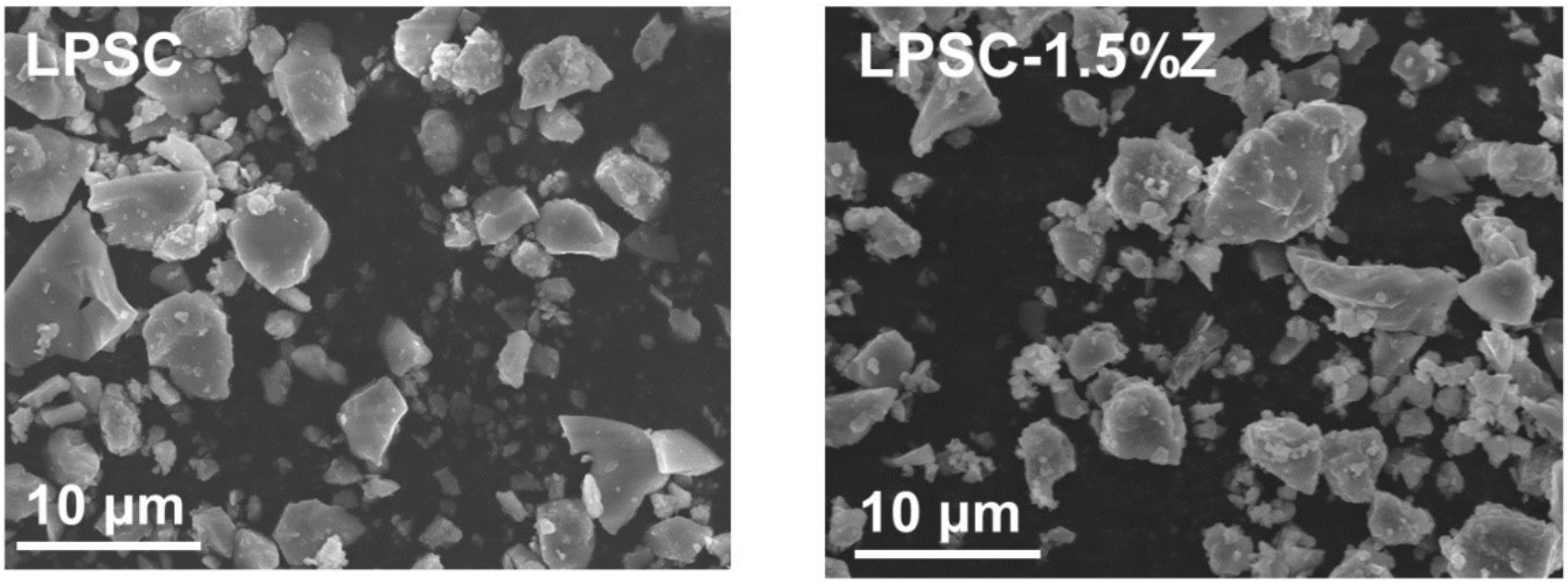

Fig. S10. SEM images of LPSC and LPSC-1.5%Z

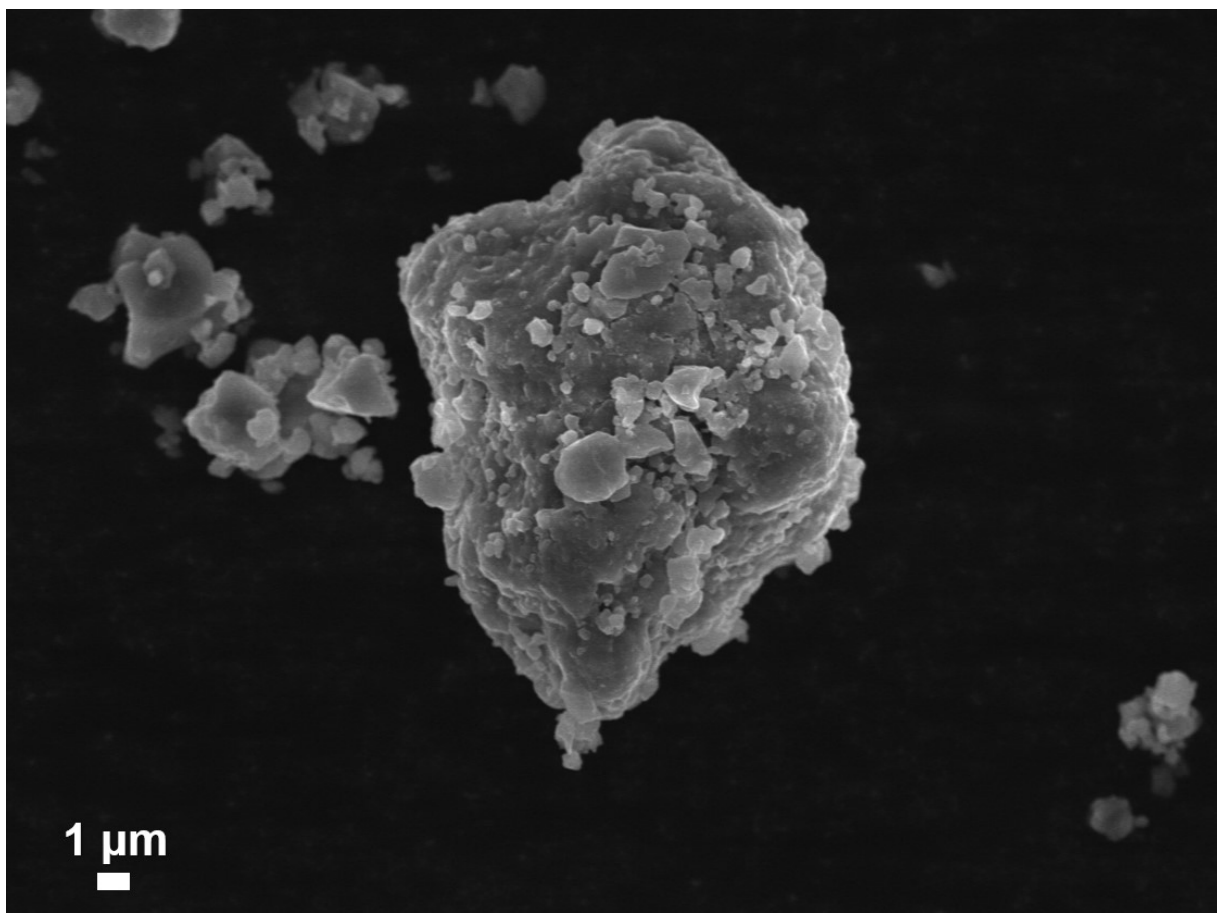

Fig. S11.SEM image of LPSC-1.5%Z

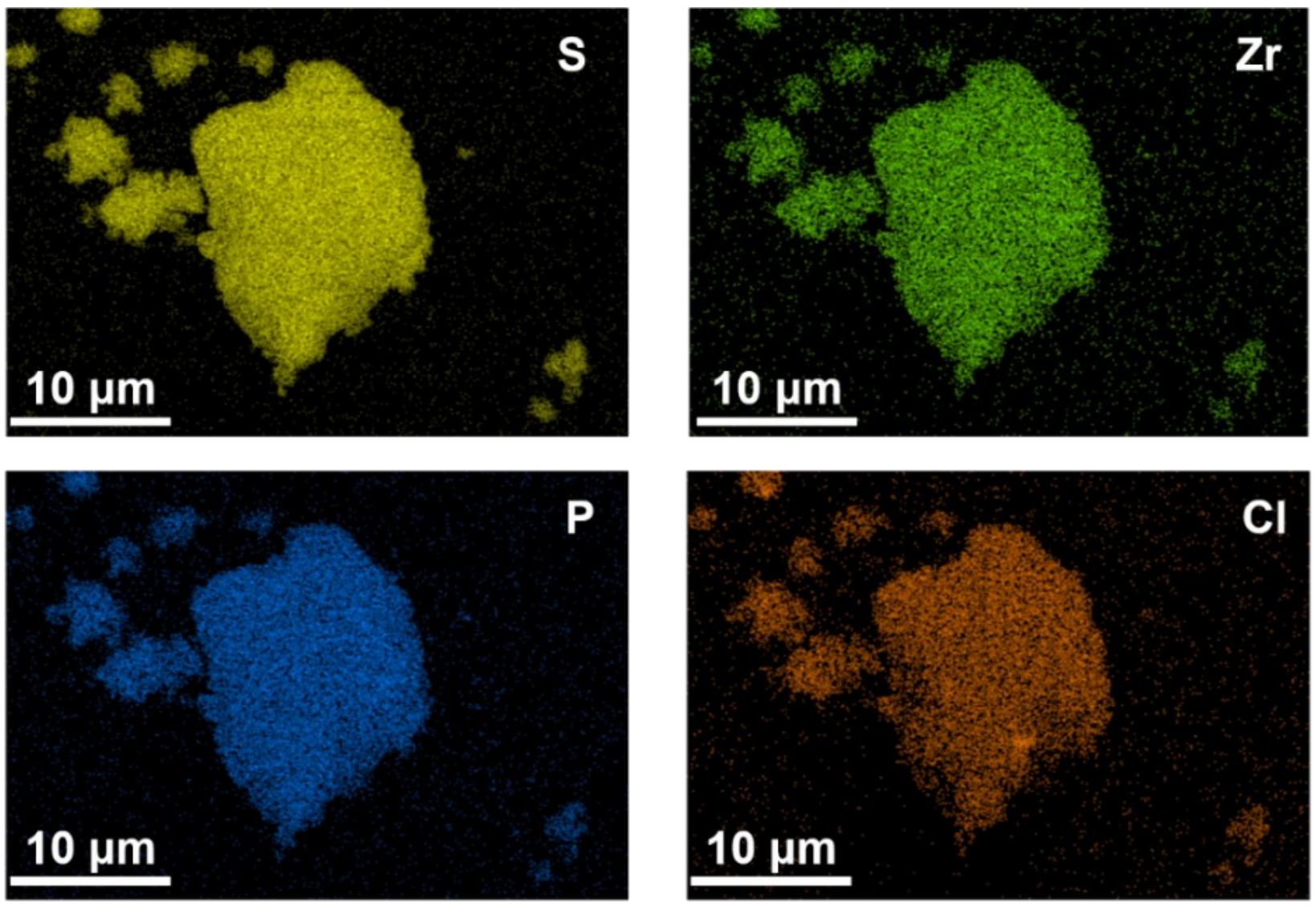

Fig. S12. EDS elemental mapping of LPSC-1.5%Z

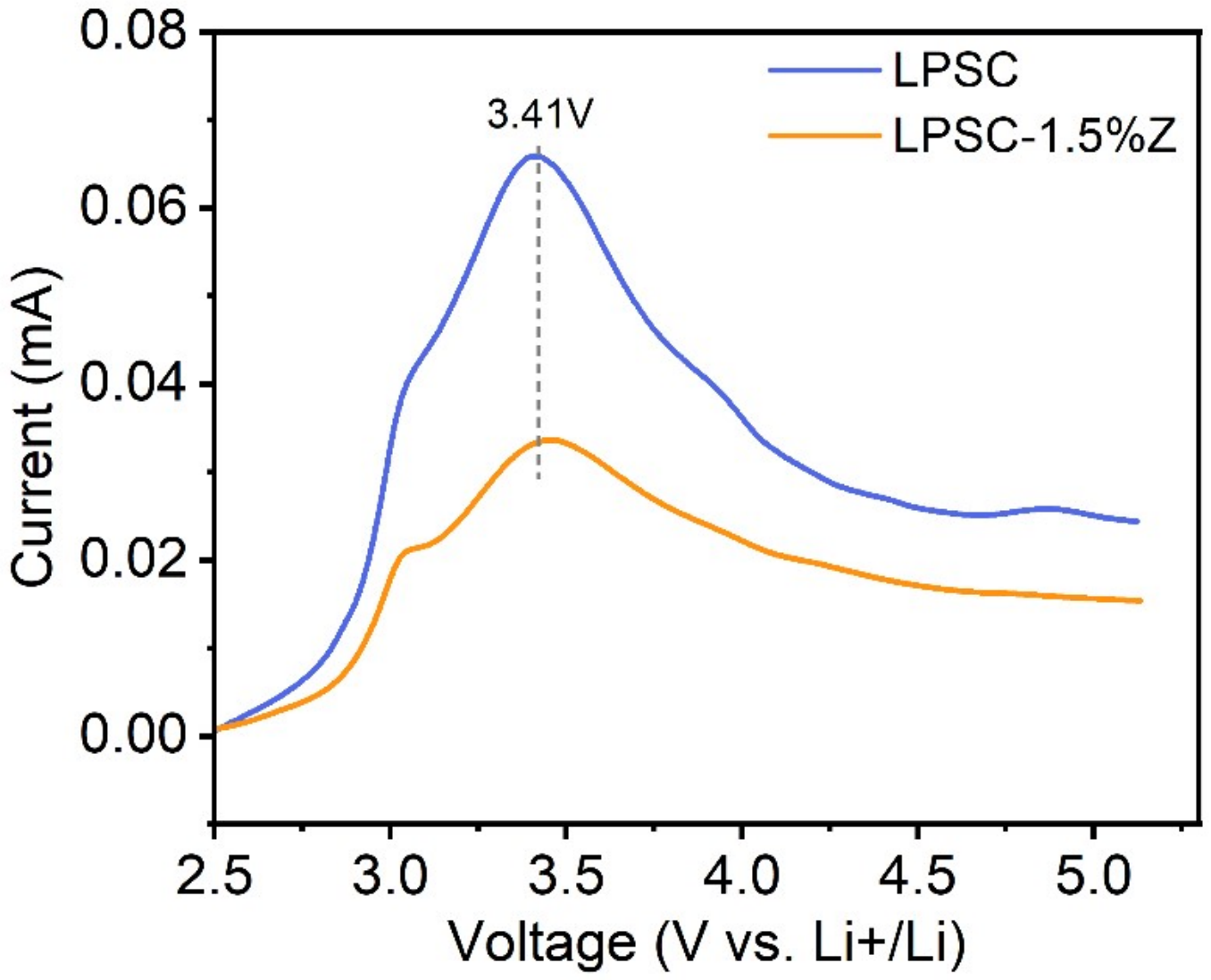

Fig.S13. LSV curves of LPSC and LPSC-1.5%Z.

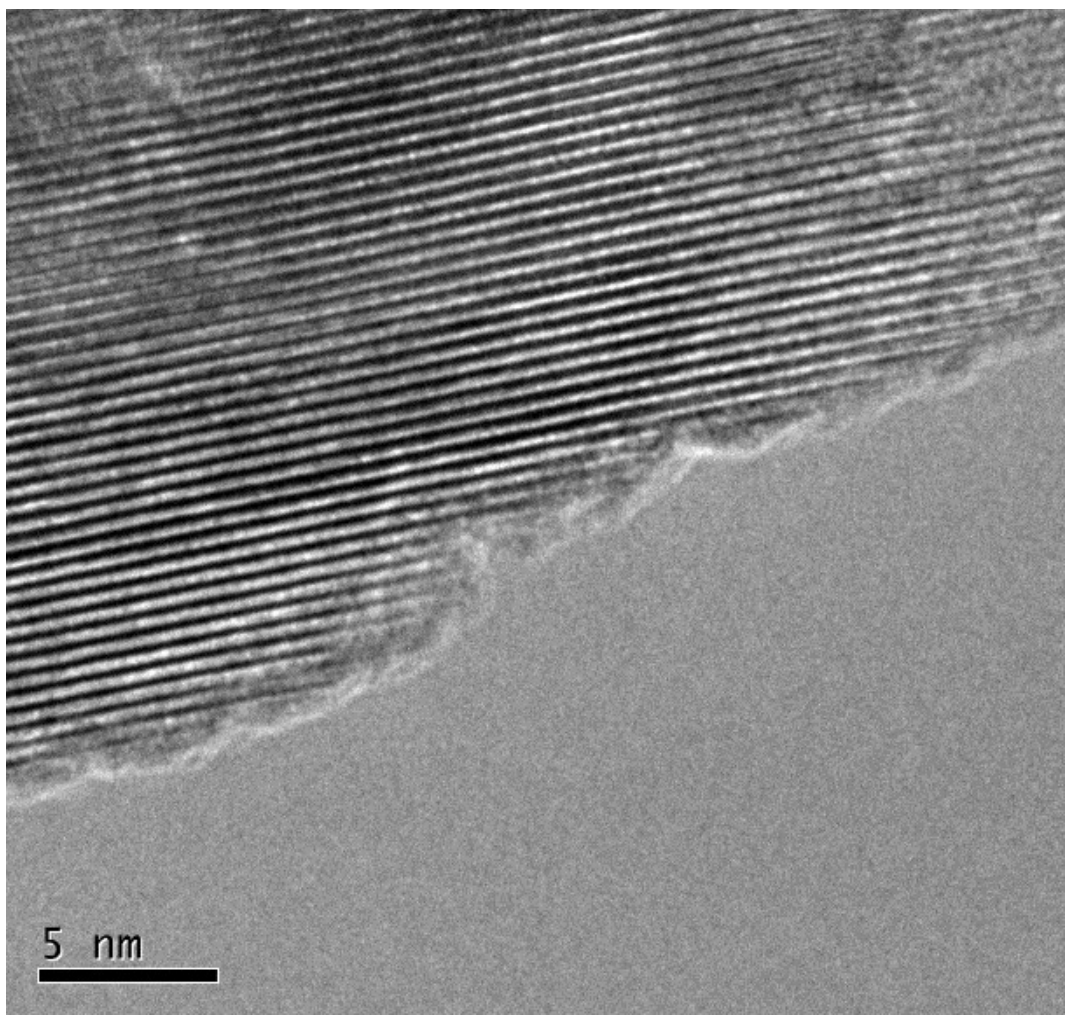

Fig. S14. TEM images of the bare NCM811 cathode

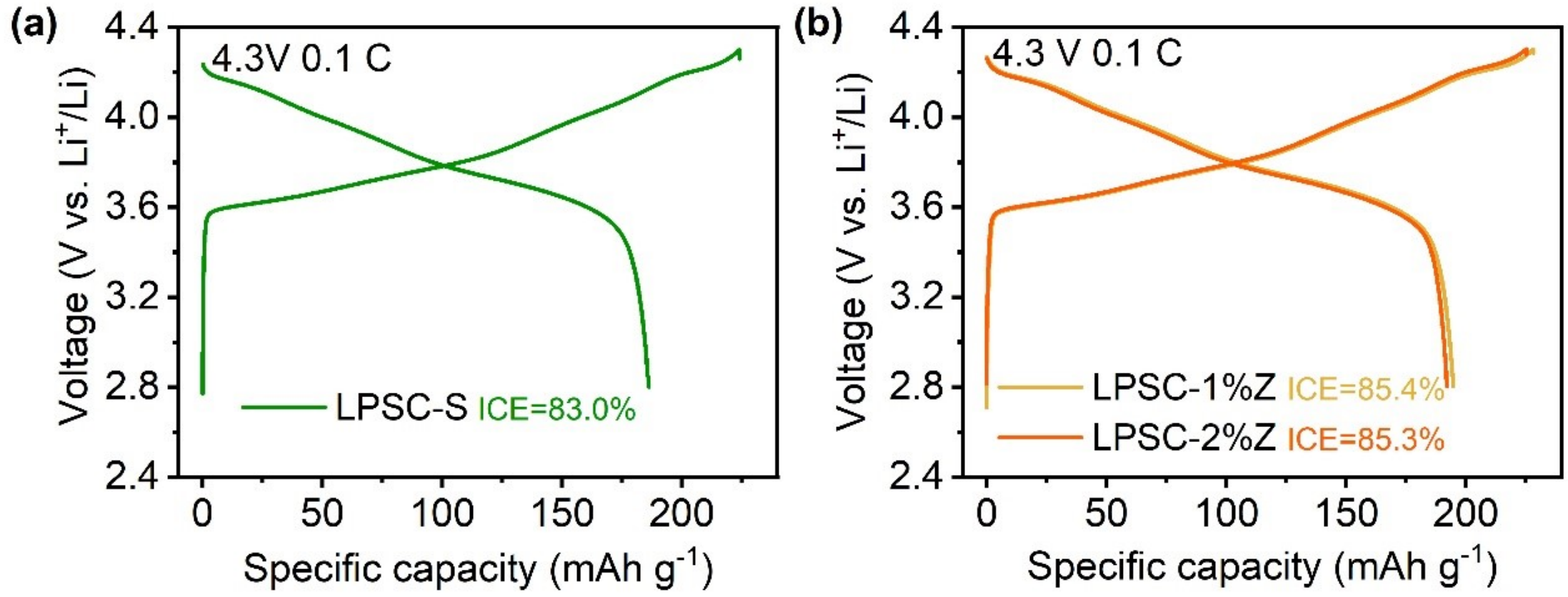

Fig. S15. Initial charge–discharge curve of (a) LPSC-S, (b) LPSC-1%Z and LPSC-2%Z

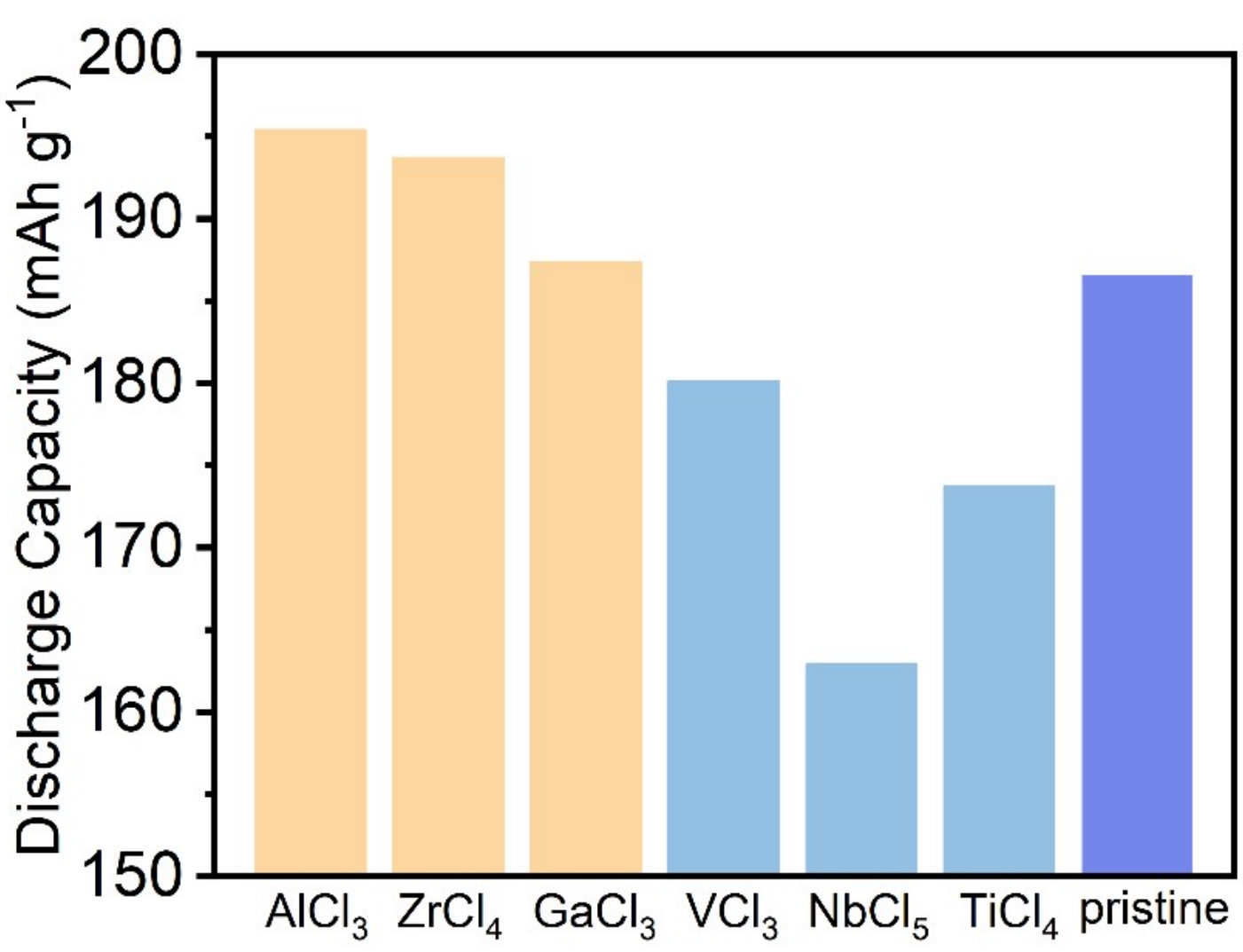

Fig. S16. Initial discharge capacities of LPSC treated with different metal halides

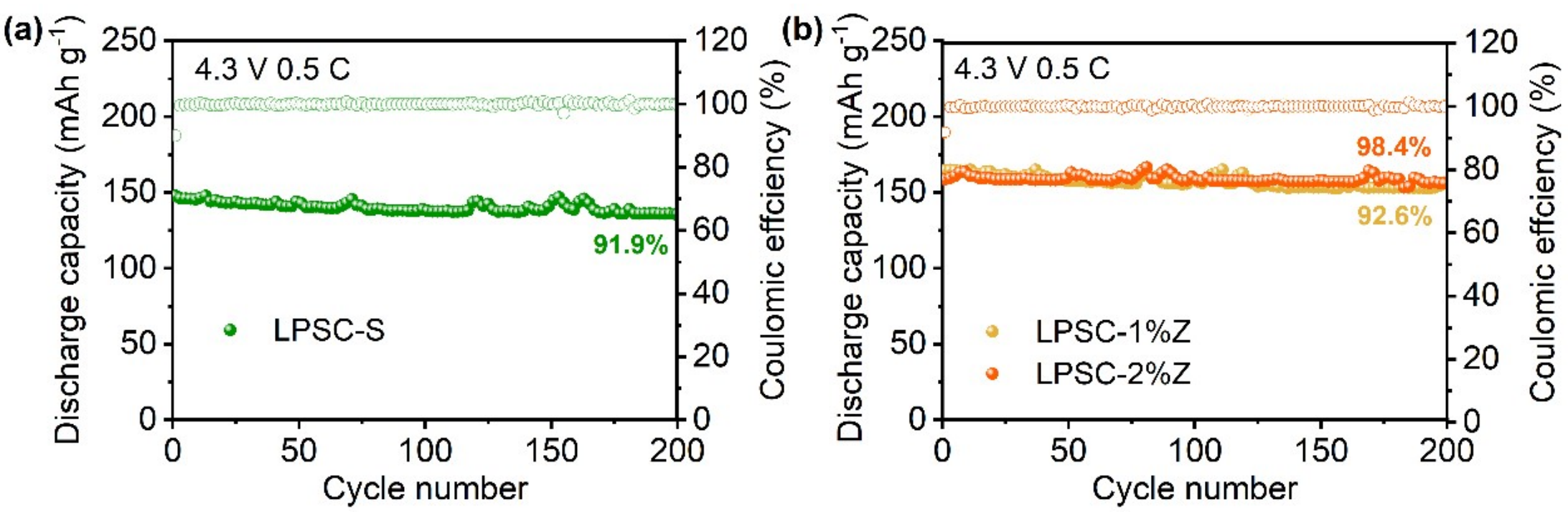

Fig. S17. Cycling performance of sintered (a) LPSC-S, (b) LPSC-1%Z and LPSC-2%Z at 0.5C for 200 cycles

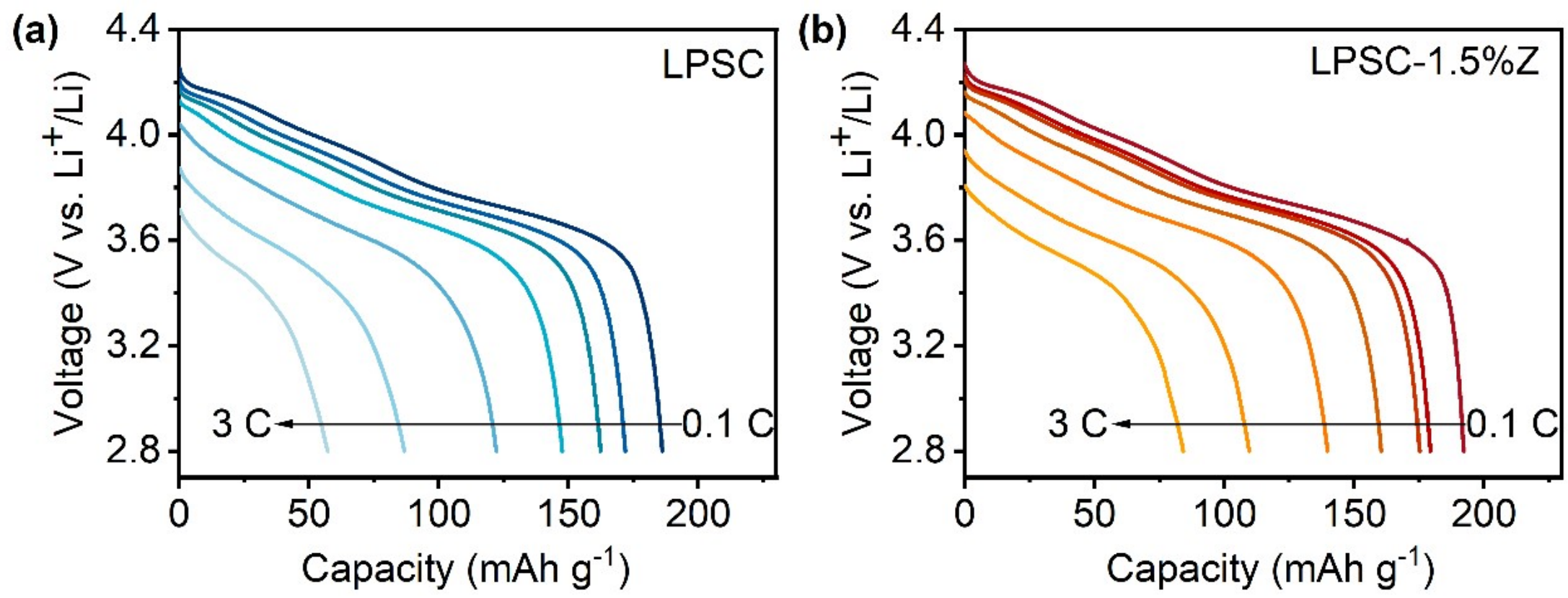

Fig. S18. Discharge curves of (a) LPSC and (b) LPSC-1.5%Z at different rates

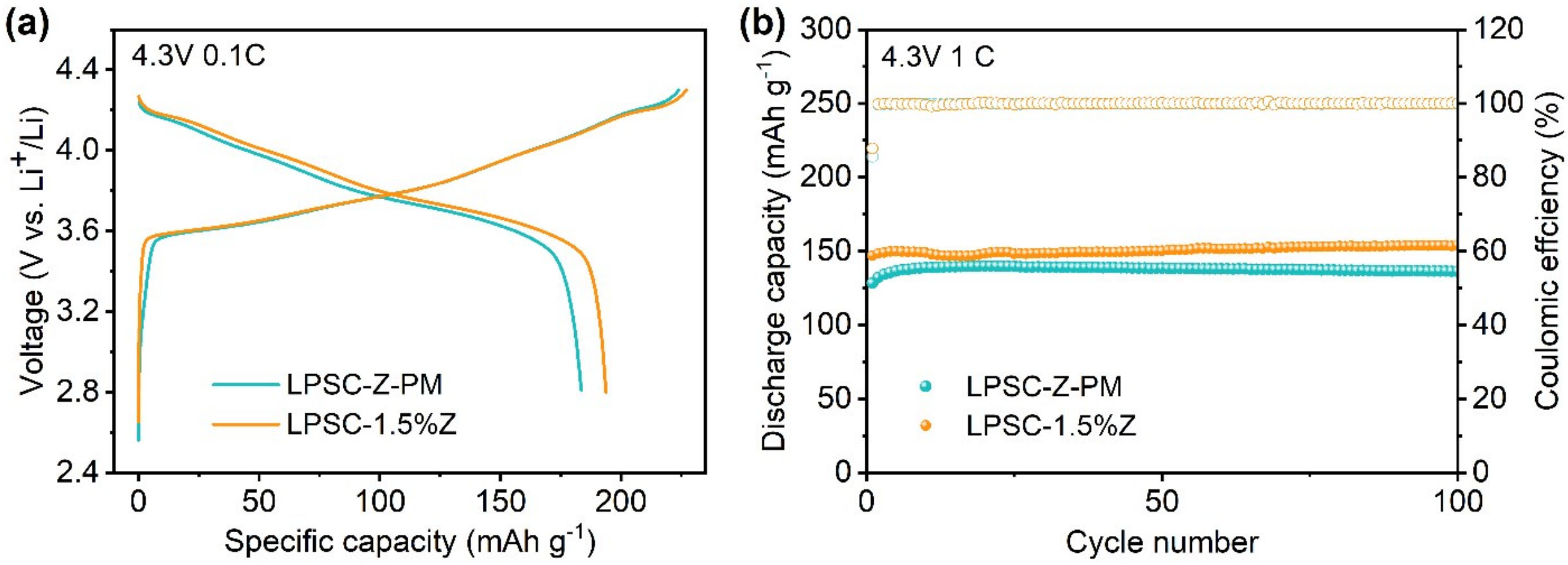

Fig. S19. (a)Initial charge-discharge curve and (b)cycling performance of the cell assembled with the LPSC-Z-PM and LPSC-1.5%Z

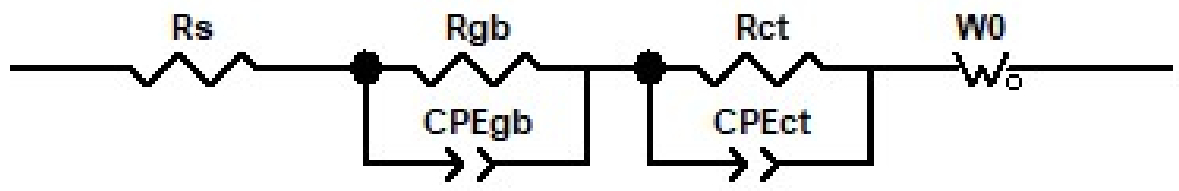

Fig. S20. Equivalent circuit schematic.

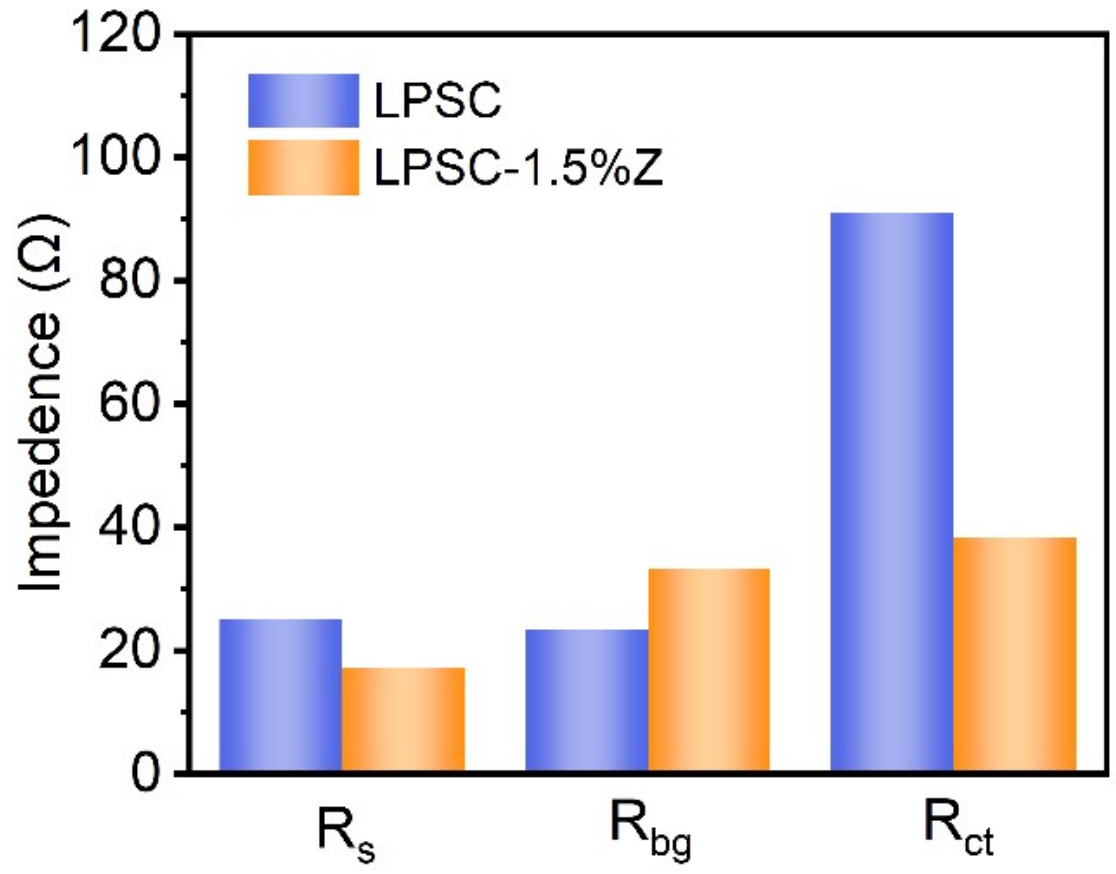

Fig. S21. Statistical analysis of impedance data for LPSC and LPSC-1.5%Z after equivalent circuit fitting.

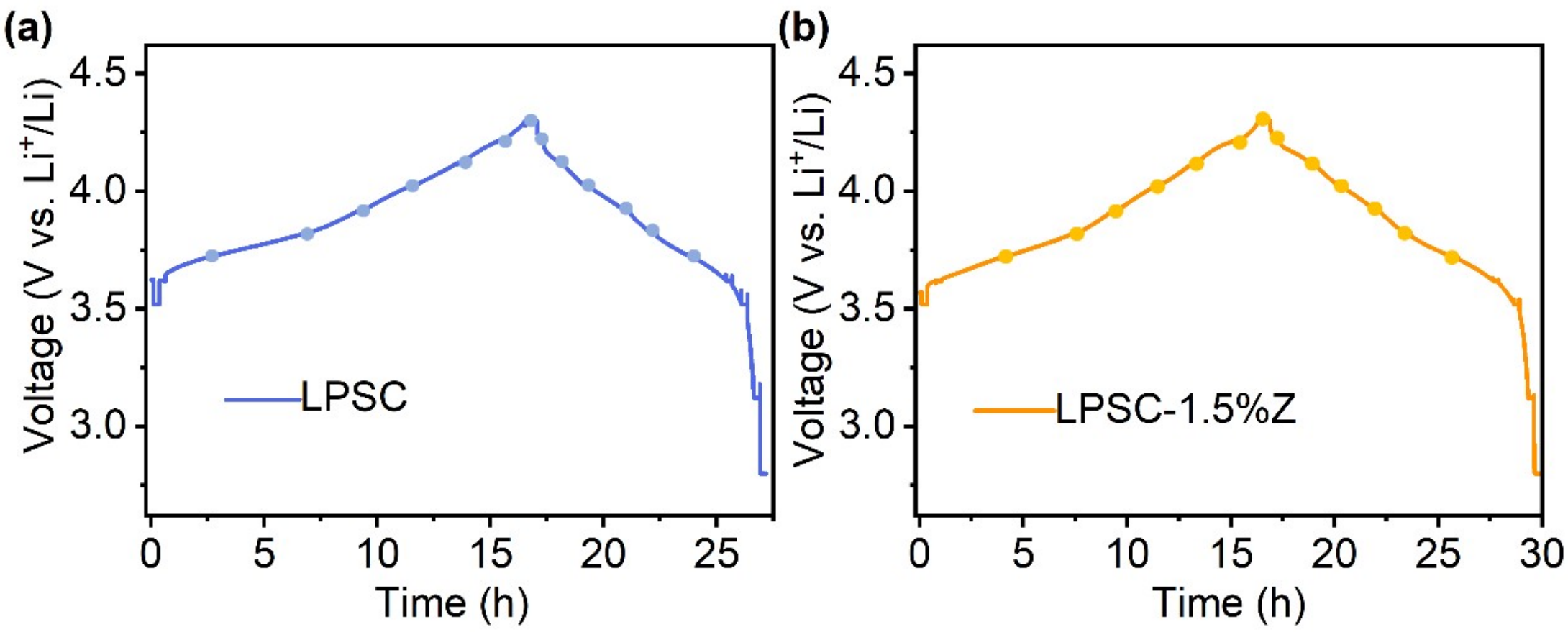

Fig. S22. Voltage-time profile for in situ EIS measurements of an NCM‖LiIn cell using LPSC and LPSC-1.5%Z at 0.1C and 30 °C

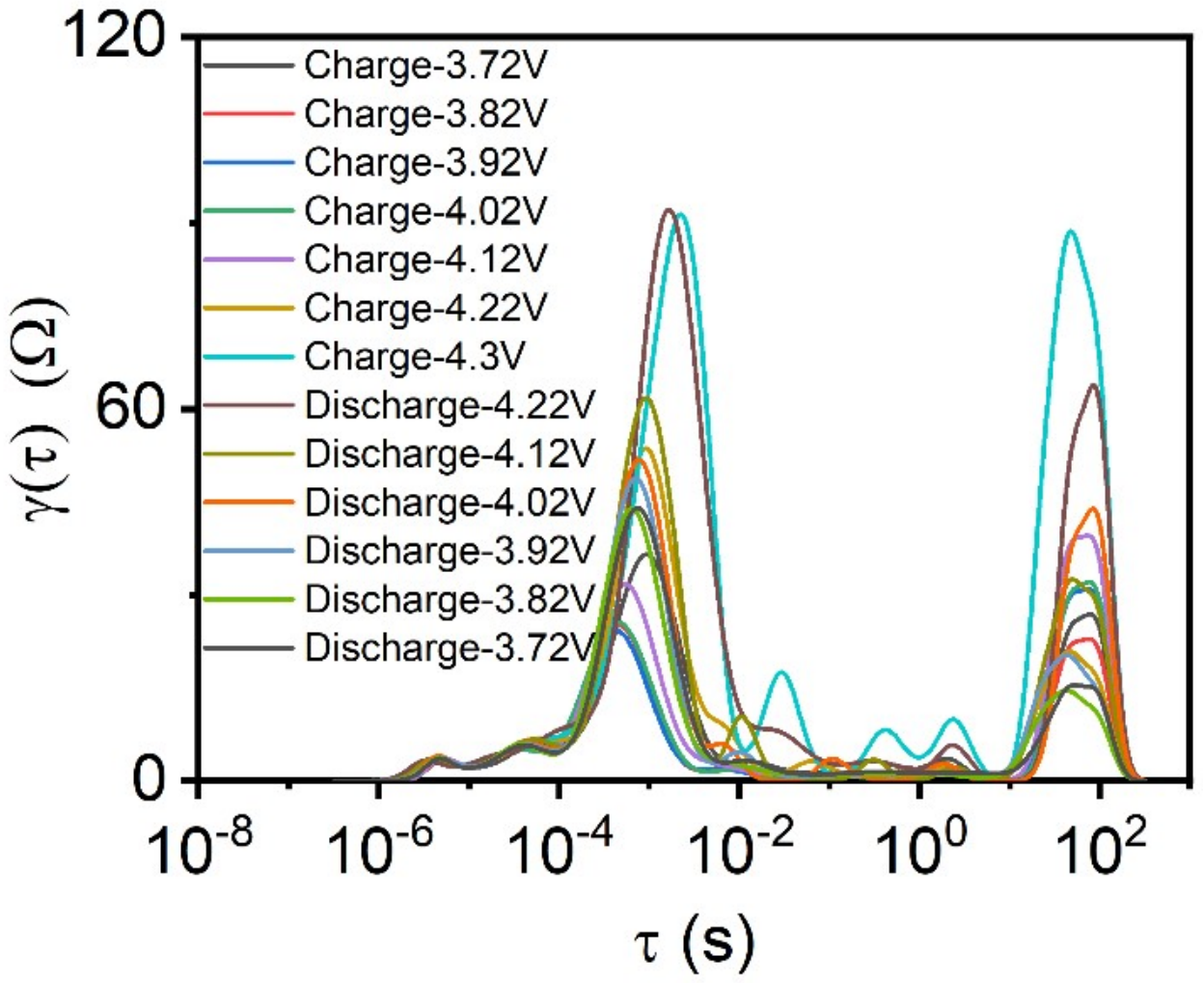

Fig. S23. DRT analysis derived from EIS of LPSC at different voltages

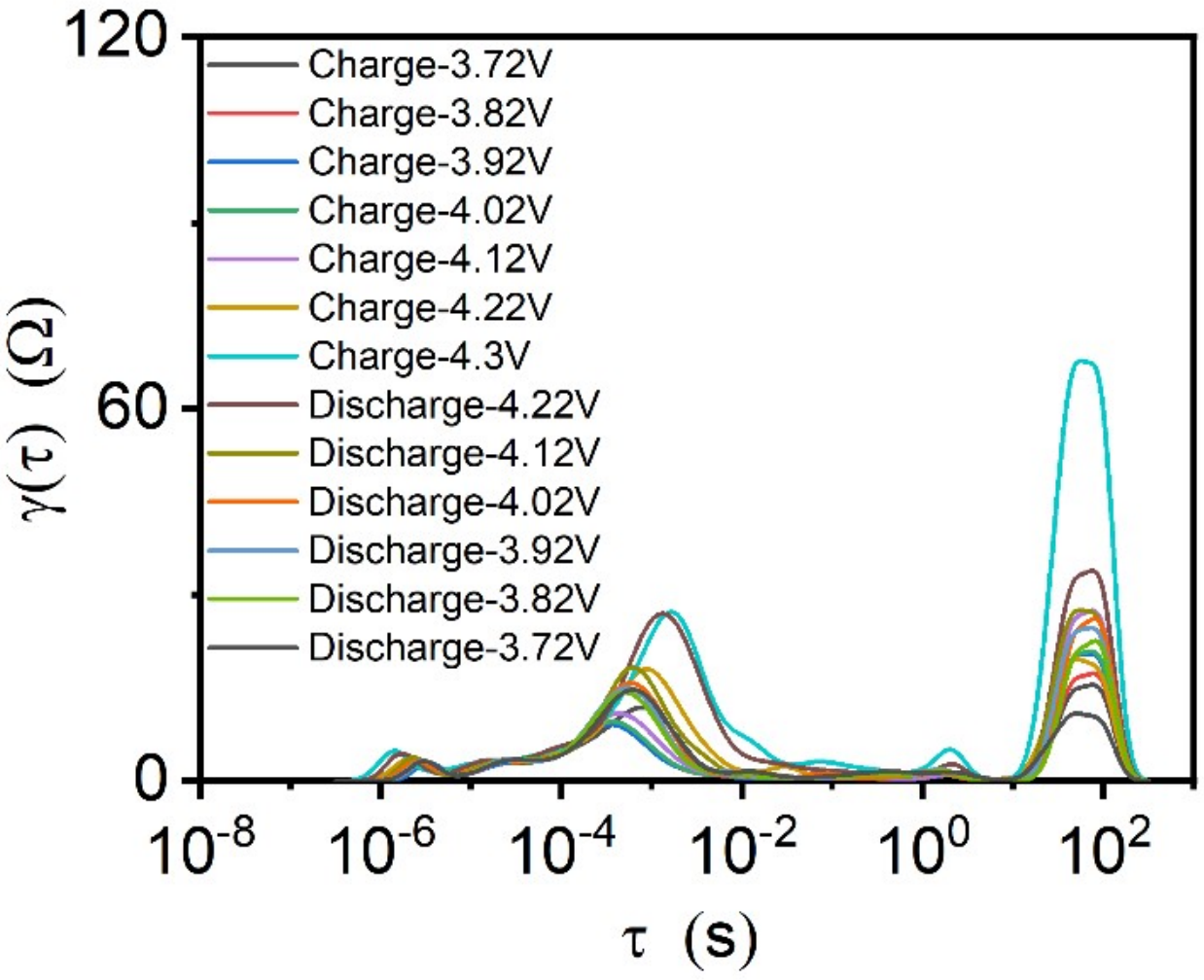

Fig. S24. DRT analysis derived from EIS of LPSC-1.5%Z at different voltages

Notes and references

1 Q. Zhu, Y. Jin, B. Zhang, W. Liu, H. Qin, L. Ming, X. Ou, Chinese Chemical Letters 2026, **37**, 111411.

2 F. Ren, Z. Liang, W. Zhao, W. Zuo, M. Lin, Y. Wu, X. Yang, Z. Gong, Y. Yang, Energy & Environmental Science 2023, **16**, 2579–2590.

3 K. Wang, Z. Liang, S. Weng, Y. Ding, Y. Su, Y. Wu, H. Zhong, A. Fu, Y. Sun, M. Luo, J. Yan, X. Wang, Y. Yang, ACS Energy Letters 2023, **8**, 3450–3459.

4 J. Aspinall, Y. Chart, H. Guo, P. Shrestha, M. Burton, M. Pasta, ACS Energy Letters 2024, **9**, 578–585.

5 C. D. Alt, S. Keuntje, I. L. Schneider, J. Westphal, P. Minnmann, J. K. Eckhardt, K. Peppler, J. Janek, Advanced Energy Materials 2024, **15**, 2404055.
